# Physiological and Epigenetic Responses of the Long‐Spined Sea Urchin *Diadema antillarum* Across a Spatiotemporal Gradient

**DOI:** 10.1002/ece3.72915

**Published:** 2026-01-12

**Authors:** Ibis T. Lopez‐Jimenez, Alex E. Mercado‐Molina, Juliet M. Wong, Jose Eirin‐Lopez

**Affiliations:** ^1^ Environmental Epigenetics Laboratory, Institute of Environment Florida International University Miami Florida USA; ^2^ University of Puerto Rico‐Bayamón Bayamón Puerto Rico; ^3^ Nicholas School of the Environment, Division of Marine Science and Conservation Duke University Marine Lab Beaufort North Carolina USA

**Keywords:** coral reef restoration, *Diadema antillarum*, DNA methylation, environmental heterogeneity, physiological plasticity

## Abstract

Following catastrophic population declines in the 1980s and 2022, the keystone herbivore *Diadema antillarum* has become a focal species for Caribbean‐wide restoration initiatives. In the present work, we combined an 11‐month field survey across four reefs on the island of Culebra, Puerto Rico, with reciprocal transplants to evaluate physiological performance and DNA methylation responses of *D. antillarum* to seawater temperature, salinity, sedimentation, and nutrient gradients. Environmental parameters varied significantly across sites and seasons (GLM, *p* < 0.01). Urchin densities were negatively correlated with sedimentation, and righting response (a proxy for neuromuscular function) slowed under elevated sedimentation. Epigenetic analyses revealed extensive DNA methylation variation clustering by season rather than site. Righting response correlated significantly with DNA methylation patterns, suggesting a role of epigenetic regulation in physiological plasticity. Surviving transplanted urchins rapidly recovered normal righting behavior, indicating individual‐level acclimatization despite ~50% transplant mortality primarily attributed to handling stress rather than environmental incompatibility. Collectively, our results suggest that restoration efforts should prioritize low‐sedimentation sites (< 30 mg·cm^−2^·day^−1^) while implementing refined handling protocols and preconditioning strategies to enhance transplant success and minimize procedural mortality in suboptimal environments.

## Graphical abstract

Following catastrophic population declines in the 1980s and 2022, the keystone herbivore *Diadema antillarum* has become a focal species for Caribbean‐wide restoration initiatives. In the present work, we combined an 11‐month field survey across four reefs in the island of Culebra, Puerto Rico, with a reciprocal transplant experiment to evaluate physiological and epigenetic responses to environmental gradients. Our results suggest that sedimentation and nutrient loading, alongside strategic population supplementation from stress‐adapted source populations, should be prioritized during coordinated habitat remediation strategies. This work provides the first comprehensive characterization of epigenetic responses in *D. antillarum*, providing molecular baselines with potential applications for species recovery programs.

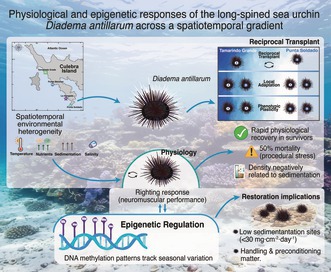

## 1 Introduction

Climate‐driven disturbances to coral reefs have been extensively studied (Goulet and Goulet 2021; Hoegh‐Guldberg et al. 2023; Jury et al. 2024), yet the responses of other key reef organisms, particularly herbivorous invertebrates, have received comparatively less attention (Pendleton et al. 2016; Przeslawski et al. 2008; Rogers 2013; Stier and Osenberg 2024). Herbivores play essential roles in maintaining reef resilience by controlling macroalgal growth (Mumby and Steneck 2008; Steneck et al. 2018). Unchecked macroalgae proliferation can dominate reef substrates and competitively exclude corals, thereby shifting ecosystems toward persistent macroalgae‐dominated states (Hughes et al. 2007). As such, understanding the environmental sensitivities of reef herbivores is crucial for effective ecosystem management.

The long‐spined sea urchin, *Diadema antillarum* , serves as a key herbivore on Caribbean reefs, controlling macroalgal overgrowth and facilitating coral recruitment (Edmunds and Carpenter 2001; Idjadi et al. 2010; H. A. Lessios 2016; Spiers and Frazer 2023). This echinoderm influences reef structure through multiple mechanisms, including preventing competitive exclusion, enhancing substrate availability for larval settlement, and contributing to nutrient cycling via bioturbation (Carpenter and Edmunds 2006; Idjadi et al. 2010; Latijnhouwers et al. 2024; Tuya et al. 2005). However, *D. antillarum* populations have declined severely due to multiple stressors (Hylkema et al. 2023; H. Lessios 1988, 2016; Levitan et al. 2023), including a catastrophic mass mortality event in the early 1980s, which decimated 94%–100% of the populations across the Caribbean, precipitating widespread shifts from coral‐ to macroalgae‐dominated reefs (Carpenter and Edmunds 2006; Hylkema et al. 2023; H. A. Lessios 2016). Since then, natural recovery has been limited, with population densities remaining well below pre‐die‐off levels (Mercado‐Molina et al. 2015; Rodríguez‐Barreras et al. 2023; Tuohy et al. 2020). A second mass mortality event in 2022, which caused widespread additional declines in already vulnerable populations (Hylkema et al. 2023; Levitan et al. 2023).

These declines have stimulated restoration programs involving outplanting of nursery‐reared urchins into degraded reefs (Hylkema et al. 2022; Pilnick et al. 2021; Williams 2016). Yet, since the demographic performance of those individuals will be importantly influenced by their physiological responses to site‐specific environmental factors, the success of these strategies will be further increased by a deeper understanding of how urchins respond to prevailing local environmental conditions (Bodmer 2019; Bodmer et al. 2017; Clemente and Hernández 2008). Understanding this capacity is vital for refining restoration strategies and identifying site‐specific constraints on urchin acclimatization and performance.

Phenotypic plasticity and acclimatization, modulated in part by epigenetic mechanisms such as DNA methylation, is increasingly recognized as a key factor mediating organismal responses to environmental stress (Eirin‐Lopez and Putnam 2019). In echinoderms, epigenetic modifications have been linked to regulation of metabolic, developmental, and immune responses (Chang et al. 2023; Ebert 1997; Xu et al. 2019), including responses to environmental stress (Han et al. 2021; Liu et al. 2023; Strader et al. 2020; Zhang et al. 2024). While numerous studies have examined DNA methylation in various sea urchin species (Bogan et al. 2023; Liu et al. 2023; Rahman et al. 2024; Strader et al. 2019, 2020), there is a lack of data for *D. antillarum* . To address this gap, we integrated physiological and epigenetic approaches to investigate how *D. antillarum* responds to variable environmental conditions. Our study combined two complementary approaches: (1) a longitudinal monitoring of seasonal changes in urchin performance across reefs with contrasting environmental profiles, and (2) a reciprocal transplant experiment designed to directly assess the degree of physiological plasticity and epigenetic reprogramming induced by relocation to novel environmental contexts. This transplant experiment explicitly tests that epigenetic mechanisms contribute to the capacity of *D. antillarum* to acclimatize to altered environments, with direct implications for restoration site selection and adaptive management.

## 2 Materials and Methods

### 2.1 Site Selection and Monitoring

**FIGURE 1:**
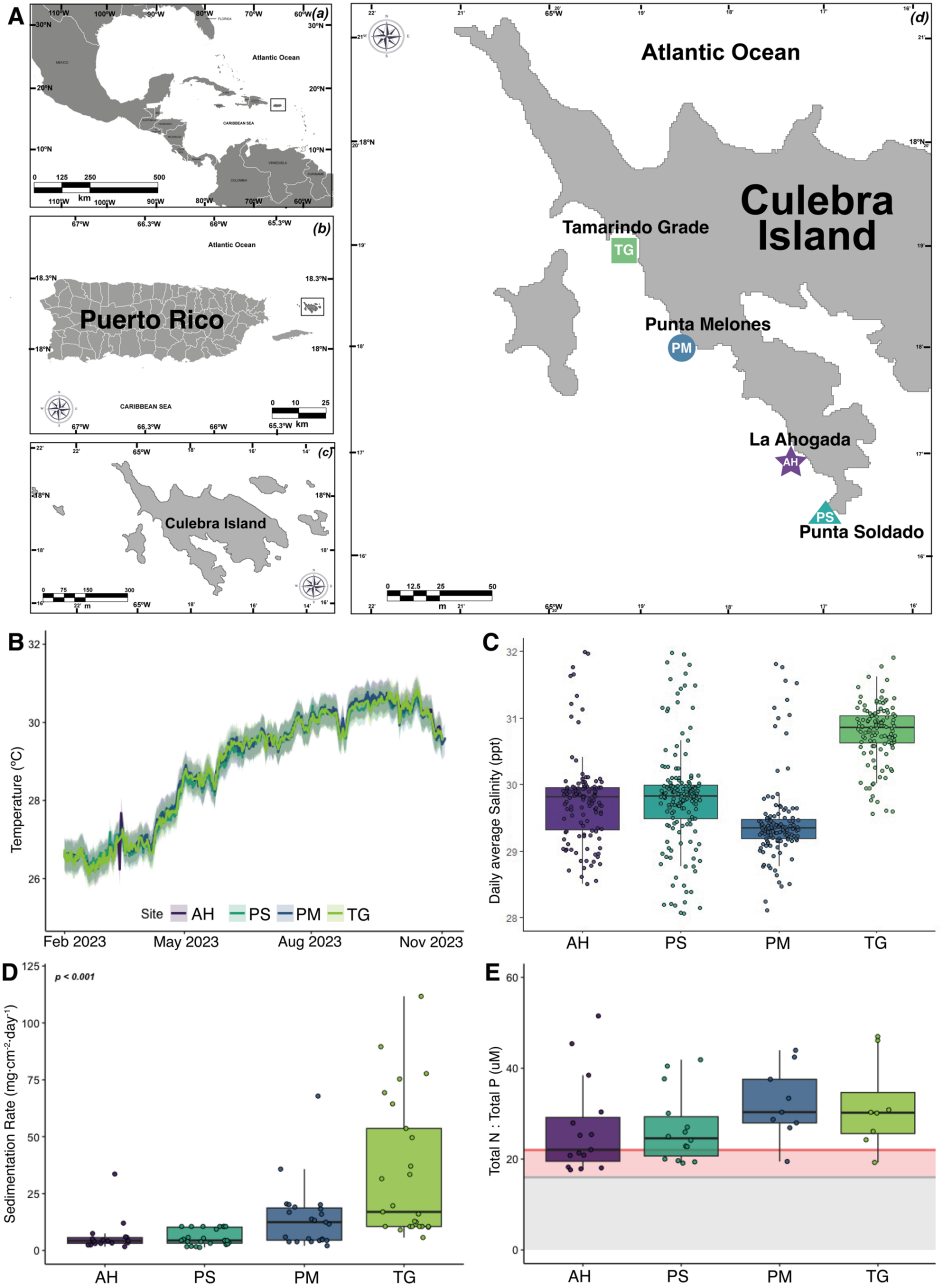
Study site overview and spatiotemporal dynamics of key environmental parameters across study sites. (A) Locations of four study sites on Culebra Island, Puerto Rico, include four sites located at Tamarindo Grande (TG; N 18.323370°, W 65.328008°), Punta Melones (PM; N 18.307029°, W 65.313742°), La Ahogada (AH; N 18.287880°, W 65.296823°), and Punta Soldado (PS; N 18.280103°, W 65.287875°). (B) Temporal evolution of water temperature throughout the initial monitoring year across all study sites. Solid lines represent daily mean values, while the surrounding shaded envelopes delineate the diurnal thermal range (daily minima and maxima). (C) Daily average of spatial patterns in salinity across the monitoring period when data was logged. (D) Spatial patterns in sedimentation rate are expressed as monthly averages for each study site. (E) Nutrient stoichiometry (total N:P ratio) across study sites. Reference thresholds include the canonical Redfield ratio (16:1 N, horizontal gray line; Redfield 1958) and the phosphorus limitation threshold (22:1, horizontal orange line; Rosset et al. 2017). The gray shaded region demarcates potential nitrogen limitation (ratios ≤ 16:1), while the orange shaded region indicates the transition zone toward phosphorus limitation (ratios between 16:1 and 22:1). In all cases, boxes indicate the median (horizontal line), interquartile range (25th–75th percentiles), and whiskers extend to 1.5 × IQR. Individual data points are overlaid to show sample dispersion.

Four study sites along western Culebra, Puerto Rico (Figure 1A), were selected based on regional conservation initiatives and the presence of *D. antillarum* . These locations were: Tamarindo Grande (TG; N 18.323370°, W 65.328008°), Punta Melones (PM; N 18.307029°, W 65.313742°), La Ahogada (AH; N 18.287880°, W 65.296823°), and Punta Soldado (PS; N 18.280103°, W 65.287875°). Sites were chosen based on historical and recent ecological data; we selected reefs with documented differences in biotic (distinct urchin density, coral, and macroalgae cover) and abiotic characteristics (García‐Sais et al. 2016; Gómez‐Andújar and Hernandez‐Delgado 2020; Rodríguez‐Barreras et al. 2015).

Environmental conditions were assessed by measuring seawater temperature, salinity, sedimentation rate, and nutrients at each site over a duration of 11 months (between January and November 2023). At each site, Onset HOBO Pendant MX Temperature loggers were deployed to monitor seawater temperature every 15 min. Salinity was monitored every hour using Onset HOBO Saltwater Conductivity/Salinity Data Loggers. Sedimentation rates were assessed by using triplicate PVC sediment traps (45 cm tall, 6.4 cm aperture diameter) deployed at each site (Otaño‐Cruz et al. 2017); deposited sediment were collected every 6 weeks. Sedimentation rate (mg·cm^−2^·day^−1^) was calculated by dividing the weight of dried sediment by the size of the trap opening and deployment duration. Nutrient levels were assessed via analysis of the macroalgae *Dictyota* sp., a common coastal bioindicator of nutrient enrichment in Puerto Rico (Todd et al. 2009). *Dictyota* were collected periodically at each site, and concentrations of total Nitrogen, total Phosphorus were quantified at the Center for Aquatic Chemistry and Environmental Nutrient Analysis Core Facility at Florida International University (FIU).

### 2.2 Population and Physiological Assessment

To evaluate environmental influences on population dynamics, *D. antillarum* individuals were counted within six permanent 20‐m^2^ belt transects per site. Surveys were conducted every 6 weeks during daylight hours to optimize visibility, ensure standardized counting conditions, and document individuals near diurnal refugia. Surveys employed thorough search protocols, including examination of cryptic microhabitats within and beneath reef structures, to maximize detection probability. Population size structure was assessed via systematic transect surveys employing morphometric protocols; individuals were classified into three size categories based on the urchin diameter: small (< 40 mm), medium (40–60 mm), and large (> 60 mm; Mercado‐Molina et al. 2015; Miller et al. 2003; Rodríguez‐Barreras et al. 2014, 2015).

Physiological performance was assessed based on the righting response (time required for an individual to flip from an inverted position to an upright position), a widely used metric of echinoderm neuromuscular function under stress (Kleitman 1941; Lawrence and Cowell 1996). Compromised or stressed urchins typically exhibit longer righting times. Test diameter (a measure of body size, influenced by age, growth rate, and environmental conditions), was measured and included as a covariate in analyses to account for size‐dependent biomechanical effects on righting performance. For up to fourteen randomly selected individuals per site per sampling occasion, righting time was recorded in triplicate (Rodríguez‐Barreras et al. 2018; Sherman 2015). Urchins were collected by scuba divers using hand tools to carefully lift and transfer them into plastic bins. Once on the boat deck, they were maintained in seawater‐filled containers with continuous aeration provided by battery‐powered aquarium aerators. Urchins were fully submerged and placed on a flat surface for righting trials and were returned to their original capture sites within one hour following physiological assessments and spine tissue sampling. Spines contribute to locomotion and feeding, serve as potential biomarkers of physiological condition (Lawrence 2013), and can be collected with minimal stress due to their regenerative capacity. Physical measurements and tissue samples were taken for up to seven individuals per site per sampling; specimens from PS were excluded due to consistently low density seen during the monitoring period (Figure S1A).

### 2.3 Reciprocal Transplant Experiments

A three‐month reciprocal transplant experiment was performed between March and June 2024 (Figure S1C). Urchins were transplanted between TG and PS, sites with contrasting nutrient and sedimentation regimes. Individuals were transported in aerated, insulated bins with oxygenated seawater. Upon arrival, sea urchins were introduced into 1 m^2^ galvanized mesh cages (*n* = 14 individuals per cage per site) and secured using local rocks and reef stones, which also provided shelter and food for the urchins while ensuring controlled exposure to new environmental conditions. Control groups (TGControl at TG and PSControl at PS), subjected to identical handling sampling, caging protocols (same as transplanted urchins), and holding five urchins each (due to low densities) at their origin sites (home reefs, TG or PS) were maintained throughout the experiment to account for potential site‐specific effects independent of transplantation stress. Both control groups (TGC and PSC) and transplanted urchins were monitored monthly.

The monitoring protocol included survival counts, righting response, and test diameter measurements. Survival rates (as SR = *N*_*f*_ /*N*_*i*_; *N*_*i*_ = initial number of urchins caged in the previous month; *N*_*f*_ = number of live urchins observed in the current month). Monthly survival rates were averaged to provide population‐level estimates of transplant success, enabling statistical comparisons across treatments and time periods. Environmental parameters (seawater temperature, salinity, sedimentation, nutrients) were recorded concurrently to evaluate habitat conditions and correlate them with righting and test diameter measurements. To investigate epigenetic responses to environmental shifts, spine tissue samples were collected from all urchins at three time points (transplant status category): immediately before transplantation (pre‐transplant, BT), mid‐experiment (during acclimatization, DA; first 2 months post‐transplant), and at the end of the experiment (post‐acclimatization AA; third month). Urchins were sampled during the transplant experiment following the same methods previously described.

### 2.4 Molecular and DNA Methylation Analyses

Molecular analyses used up to three spine tissue samples collected from each *D. antillarum* individual (monitoring and transplant experiment). Spine samples were preserved in DNA/RNA Shield (Zymo) and stored at −80°C for DNA methylation analysis.

Genomic DNA (84 samples from the monitoring period and 89 samples from the transplant experiment) was extracted using the Zymo Research Quick‐DNA Miniprep Kit with an additional vacufuge (Eppendorf Vacufuge Plus) step to enhance extraction purity. DNA quality was checked by spectrophotometric analysis (NanoVue) and gel electrophoresis. Concentration was quantified using a Qubit dsDNA Broad Range Assay and standardized to 36.4 ng/μL.

Global DNA methylation patterns were assessed using a methylation‐sensitive amplified polymorphism (MSAP) technique (Reyna‐López et al. 1997), which targets 5′‐CCGG‐3′ motifs across the genome. This approach distinguishes four categorical methylation states across multiple loci, enabling broad assessment of epigenetic modifications without requiring a reference genome (Pérez‐Figueroa 2013). MSAP is particularly advantageous for ecological studies involving large sample sizes, offering robust detection of genome‐wide methylation changes in response to environmental variation (Rodríguez‐Casariego et al. 2020; Rodriguez‐Casariego et al. 2022).

For the MSAP procedure (Text S1), genomic DNA underwent parallel digestion using EcoRI (0.5 U) and the isoschizomers *Hpa*II and *Msp*I (0.2 U each), which differ in their CpG methylation sensitivity. Double‐stranded adapters were ligated to the fragments, followed by two rounds of PCR: a pre‐selective PCR (primers with one selective nucleotide) and a selective PCR (two selective nucleotides and selective amplification; Table S1). PCR products were analyzed on an ABI Prism 310 Genetic Analyzer.

### 2.5 Data and Statistical Analyses

Sampling periods were classified as Dry (January–April) or Wet (May–November) season based on local hydroclimatic patterns. Site and seasonal effects on seawater temperature (°C), salinity (ppt), sedimentation (mg·cm^−2^·day^−1^), and nutrient concentrations (total N, total P, N:P ratio [μM]) were assessed via generalized linear models (GLMs). For GMLs, distributions (Gaussian, Gamma, and Inverse‐Gaussian) were selected based on AIC (for seawater temperature and salinity) and AICc (for sedimentation and nutrients); the best‐fitting model informed subsequent ANOVA. Pairwise comparisons were conducted using estimated marginal means (*emmeans* package). Nutrient imbalance was defined by N:P ratios > 16 (Redfield 1958) and > 22 (P‐limitation threshold; Rosset et al. 2017), and their frequencies were compared across sites/seasons using proportion tests.

ANOVAs (Two‐Way ANOVA and One‐Way ANOVA when required) tested for site differences in *D. antillarum* density, size (from transect surveys), test diameter (from collected individuals), and righting response, while generalized linear models (GLMs) also identified environmental predictors of these traits. PERMANOVA and PERMDISP (*adonis*, *betadisper* functions) tested physiological metrics (righting time and test diameter) across sites and seasons. Post hoc TukeyHSD and pairwise PERMANOVA were used to identify group differences. Variance partitioning and RDA quantified the influence of site and season on physiology. Log(*x* + 1)‐transformed data were used where assumptions of normality were violated. Effect sizes were estimated as *η* ^2^ (Cohen 1973) via the *effectsize* package, and estimated marginal means (*emmeans*) were used to conduct pairwise comparisons. Spearman's rank correlation coefficients were calculated to assess relationships between test diameter and righting‐response time, with significance evaluated at *α* = 0.05.

Epigenetic data (DNA Methylation) was analyzed via PERMANOVA, PCA, and dbRDA. Shannon's diversity index (SDI, Shannon, 1948) quantified overall epigenetic heterogeneity within methylation‐susceptible loci, incorporating frequency distributions of distinct DNA methylation states across genomic regions of interest. PCA loadings were correlated with environmental variables. MSAP fragment patterns across individuals were scored to determine the DNA methylation states. Fragment presence/absence (binary matrix) was scored using GeneMapper v3.7., considering peaks between 50 and 1500 bp, with signal intensity > 40 relative fluorescence units (RFU) and present in ≥ 15% of samples. Methylation states were classified based on the amplification pattern of *Hpa*II/*Msp*I digests as non‐methylated (NMT; 1/1; signal present in both digests), hemimethylated (HMM; 1/0; signal present in one digest but absent in the other, indicating methylation on one DNA strand), or internal cytosine‐methylated (ICM; 0/1; signal present only in MspI digest, indicating CpG methylation) via the *msap* package. Loci exhibiting no amplification in either enzymatic digestion (0/0) could indicate either hypermethylation (HPM) or the absence of the target due to genetic variation. We conservatively classified 0/0 patterns as hypermethylated loci and included them in subsequent statistical analyses (Rodríguez‐Casariego et al. 2020). Only methylation‐susceptible loci (MSL) were retained (Herrera and Bazaga 2010). Discriminant analysis of principal components (DAPC; Jombart et al. 2010) was performed to further examine differences in DNA methylation profiles between seasons and transplant categories and identified the loci most contributing to these differences for each group differentiation. Clustering (k) was based on PERMANOVA and post hoc tests, retaining k −1 PCs and two discriminant axes (Thia 2023).

To link epigenetics and physiological performance, log‐transformed physiological metrics were used in dbRDA to explain methylation variation. Physiological plasticity was related to epigenetic change via PCoA centroid distances for each site‐timepoint group, and their pairwise Euclidean distances were correlated using Spearman coefficients. These analytical approaches were consistently applied across both the 11‐month monitoring period and the controlled transplant experiment. All data analyses were performed using RStudio version 12.1.

## 3 Results

### 3.1 Environmental Parameter Dynamics Across Study Sites

Environmental parameters (seawater temperature, salinity, sedimentation rate, and nutrients) exhibited pronounced spatiotemporal variation across sites (Figure 1). Seawater temperature revealed distinct temporal dynamics across sites (GLM, *p* < 0.01; Figure 1B), with maxima during the wet season and minima in the dry season (Figure S2A). From early 2023, water temperatures increased, peaking in August–September before declining toward year‐end. Salinity displayed distinct seasonal patterns across sites (GLM, *p* < 0.001), with wet season conditions reduced relative to dry season levels (Figure 1C; Figure S2D). Site‐specific responses varied considerably: PM exhibited the most pronounced seasonal decline in salinity, while TG maintained relatively stable conditions across temporal scales.

Sedimentation rate varied markedly between sites (GLM, *p* < 0.001; Figure 1D). TG showed the highest rate (mean ± SD, 33.4 ± 8.9 mg·cm^−2^·day^−1^), PM showed intermediate levels (14.3 ± 5.2 mg·cm^−2^·day^−1^), while AH and PS showed lower rates (6.2 ± 2.8 and 3.6 ± 1.5 mg·cm^−2^·day^−1^, respectively). Nutrient concentrations (total N, total P) did not vary across sites (GLM, Sites *p* > 0.05; Figure S2H,K). However, distinct seasonal patterns in nutrient availability were evident (GLM, Sites *p* < 0.05; Figure S2J,L). N:P exceeded the Redfield ratio (16:1) and differed marginally across sites and seasons (GLM, Sites *p* = 0.098 and seasons *p* = 0.001, Figure 1E). PM and TG exhibited greater within‐site nutrient variability and showed higher proportions exceeding the threshold for phosphorus limitation (22:1) (Rosset et al. 2017) compared to AH and PS.

### 3.2 *Diadema antillarum* Population Structure and Physiological Status

**FIGURE 2:**
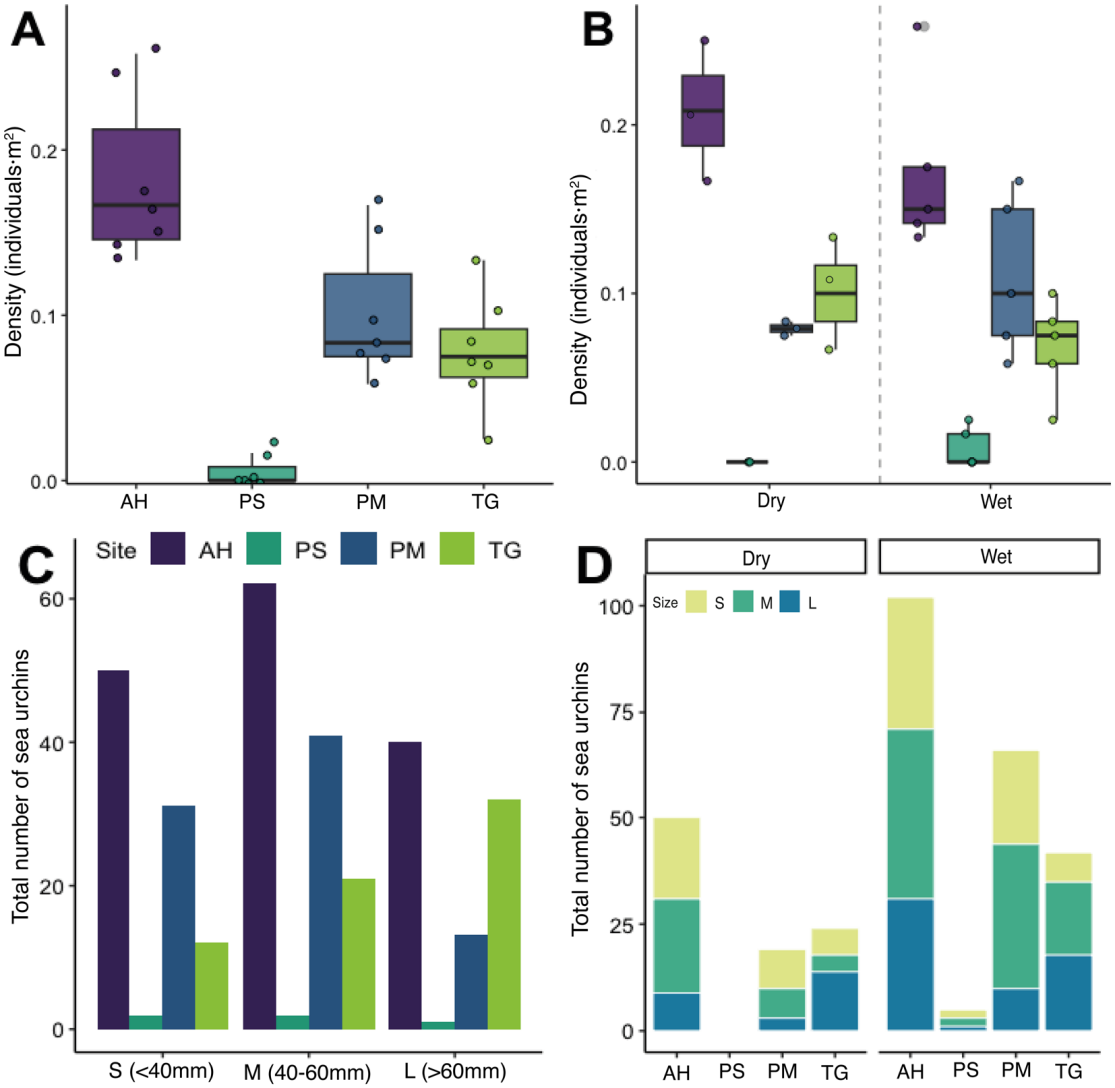
*Diadema antillarum* population density and size structure. (A) Spatiotemporal variation in urchin population density (individual·m^−2^) across study sites. (B) Seasonal comparison of mean urchin densities between the dry (January–April) and the wet (May–November) seasons. (C) Size‐frequency distribution of urchin populations across study sites during the 11‐month monitoring period. (D) Seasonal comparison of size‐frequency distribution between dry (January–April) and wet (May–November) periods. For panels A and B, boxplot elements indicate the median (horizontal line), interquartile range (25th‐75th percentiles), and whiskers extend to 1.5 × IQR. Individual data points are overlaid to show sample dispersion.

A total of 308 live *D. antillarum* individuals were recorded across sites and sampling periods (mean density ± SD, 0.092 ± 0.073 ind·m^−2^). Densities varied significantly among sites and seasons (ANOVA, both *p* < 0.05). The highest sea urchin densities occurred at AH and PM, and the lowest at TG and PS (Figure 2A; Table S2). Seasonal changes were higher at AH and TG than PM and PS (Figure 2B). Size‐frequency distributions showed a predominance of medium and larger individuals (> 40 mm) across sites (Figure 2C); overall, ~30.94% of individuals were < 40 mm, ~41.04% between 40–60 mm, and ~28.01% > 60 mm. Significant inter‐site size differences were observed (ANOVA, *p* < 0.001; Figure 2C), with size distributions exhibiting seasonal changes throughout the sampling period (Figure 2D). Significant differences in mean test diameter were observed between sites and seasons (ANOVA, site *p* < 0.001, season *p* < 0.001). TG was dominated by larger individuals (> 60 mm), while AH and PM were characterized by medium‐sized individuals (40–60 mm; Figure 2C,D; Figure S3C,D).

**FIGURE 3:**
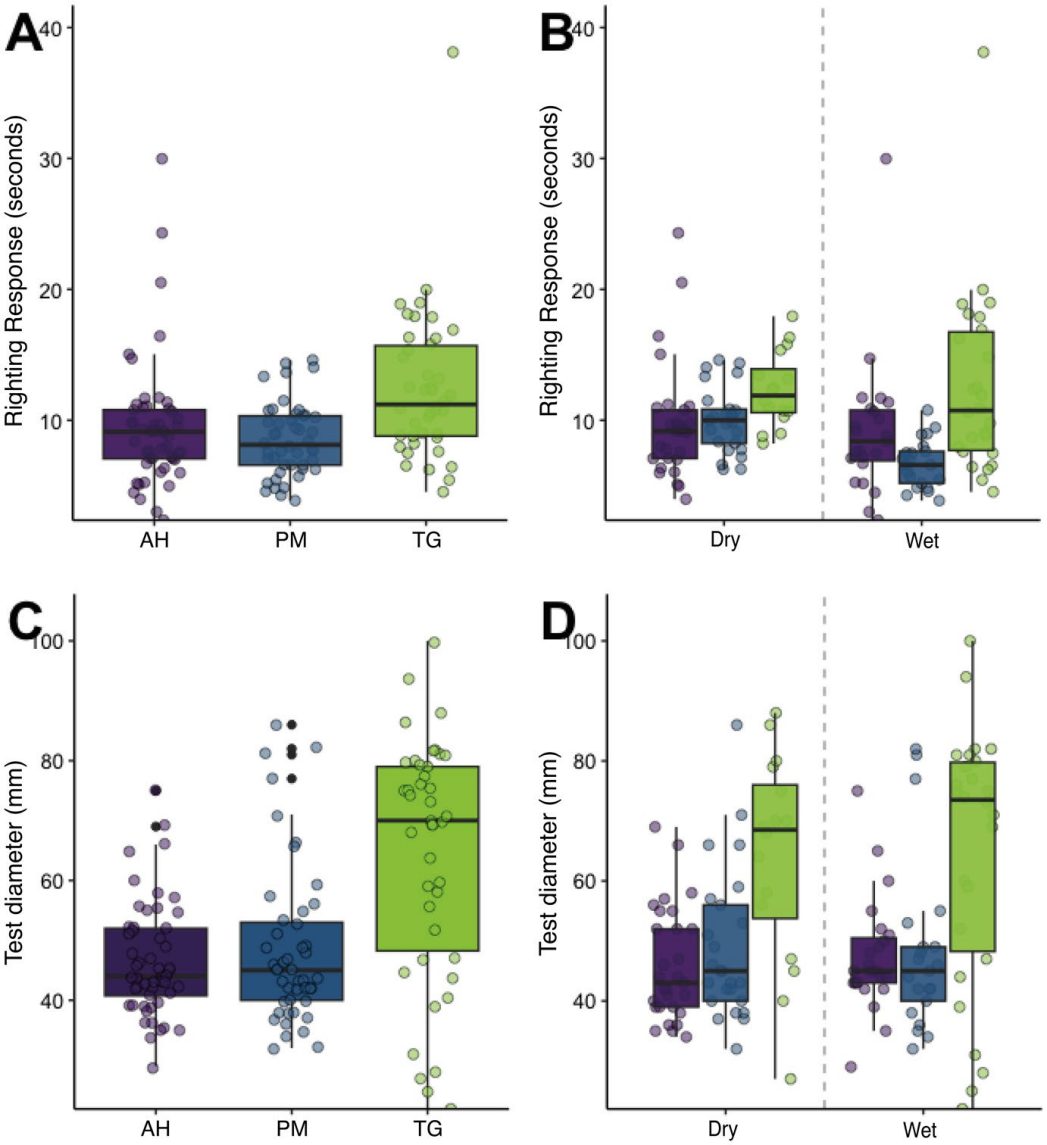
Physiological performance metrics of *D. antillarum* across spatiotemporal gradients. (A, B) Spatiotemporal analysis of righting response times (seconds) in *D. antillarum* populations across study sites and seasons during the first monitoring year, with temporal progression represented through sequential sampling periods. (C, D) Test diameter measurements in *D. antillarum* specimens across study sites throughout the first monitoring period (11 months), with temporal variation across sampling intervals. Boxplot elements indicate the median (horizontal line), interquartile range (25th–75th percentiles), and whiskers extend to 1.5 × IQR. Individual data points are overlaid to show sample dispersion.

The physiological performance of the urchins was evaluated based on measurements of righting response. The mean righting response time was 10.062 ± 4.861 s (mean ± SD) across all samples. Righting times differed significantly between sites and seasons (ANOVA, site *p* = 0.007, season *p* = 0.008; Figure 3A,B). Urchins at AH and PM showed the fastest righting responses, while TG showed the slowest in both seasons (Figure 3B). Seasonal effects differed: PM performed better (shorter righting times) during the wet season, and AH during the dry season. Righting time was positively correlated with test diameter (Spearman's *ρ* = 0.30, *p* < 0.001; Figure S4A), where larger urchins tended to take slightly longer to right themselves.

Multivariate analysis of physiological performance (i.e., righting time) showed significant differences across sites (PERMANOVA, *p* = 0.001) and seasons (PERMANOVA, *p* = 0.002; Figure S5B, Tables S3 and S4). Redundancy analysis showed that site and season together explained 22.9% of physiological variance (site = 12.2% and season = 10.7%, *p* = 0.001). Environmental factors collectively explained ~99% of physiological variance (RDA1 = 53.59%, RDA2 = 1.26%), where higher seawater temperature and salinity were positively associated with faster righting (i.e., shorter response times), whereas elevated sedimentation and higher N:P ratios were associated with slower righting responses (Figure S6A).

### 3.3 DNA Methylation Patterns Across Environmental Gradients

**FIGURE 4:**
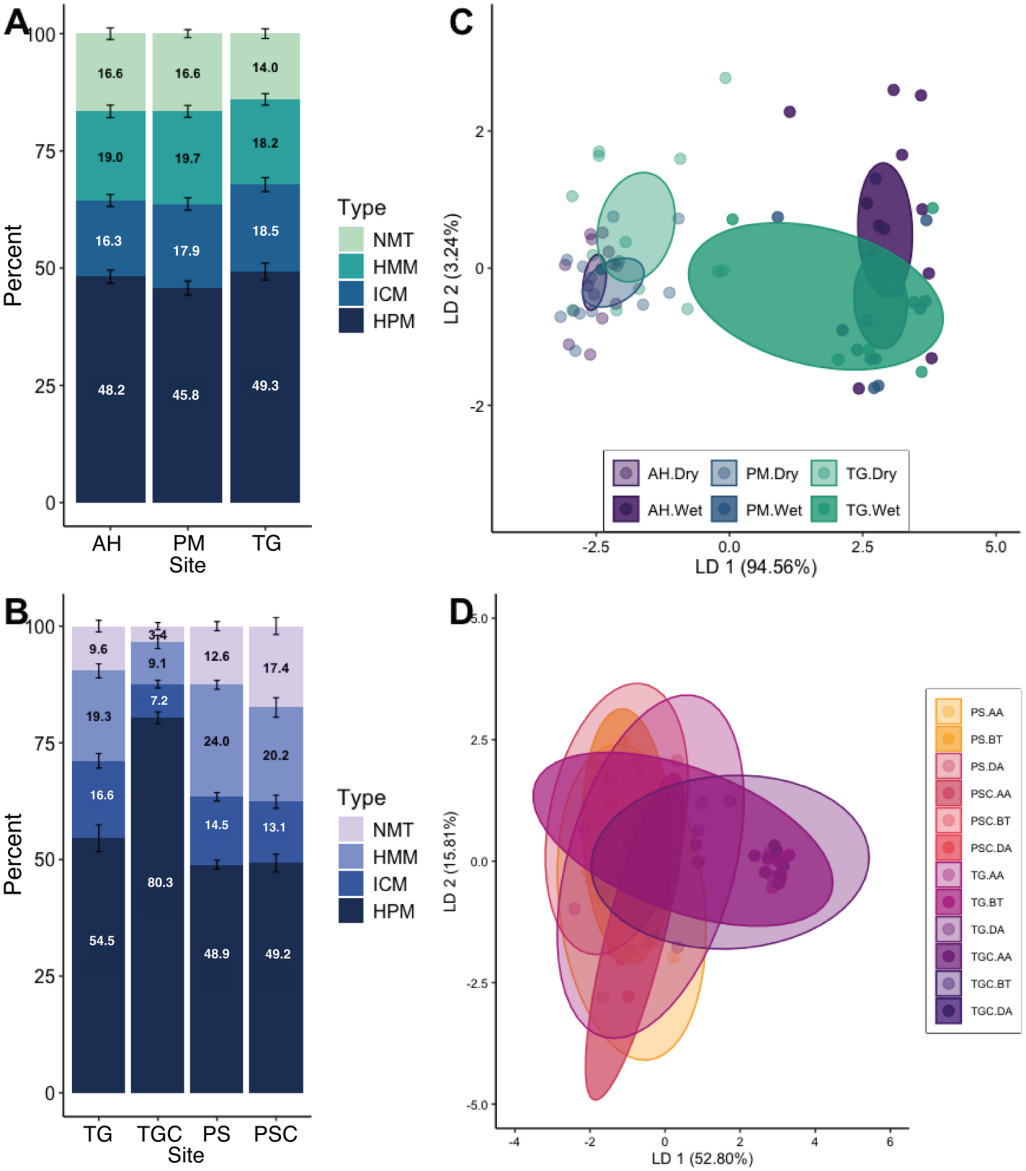
Epigenetic landscape and DNA methylation patterns in *D. antillarum* across spatiotemporal gradients in monitoring (A–C) and transplant experiment (B–D). (A, B) DNA methylation profile analysis reveal distinct epigenetic signatures across study sites. The distribution of DNA methylation states—non‐methylated (NMT), hemi‐methylated (HMM), internally cytosine‐methylated (ICM), and hypermethylated (HPM)—is represented across polymorphic loci susceptible to DNA methylation modification. Error bars represent ± SE. (C, D) Multivariate epigenetic differentiation through Discriminant Analysis of Principal Components (DAPC) with a priori classification based on statistically significant site‐specific and seasonal distinctions. In Panel C, seasonal variation is indicated by light color (dry season, January–April) and solid colors (wet season, May–November). Elliptical boundaries represent 95% confidence intervals for site‐by‐season combinations. In panel D, transplant destination sites, with elliptical boundaries delineating 95% confidence intervals for site‐by‐status combinations (AA, Post‐acclimatization/final phase; BT, Pre‐transplant; DA, during acclimatization; PSC, Punta Soldado Control; TGC, Tamarindo Grande Control).

Epigenetic analyses of DNA methylation by means of MSAP identified 294 methylation‐susceptible loci (MSL) in *D. antillarum* , including 287 loci (98%) exhibiting polymorphism. The overall epigenetic diversity within methylation‐susceptible loci (Shannon's diversity index, SDI) was 0.472 ± 0.151 (Mean ± SD). Hypermethylation (HPM) predominated among methylation states, followed by hemi‐methylation (HMM) and internal cytosine methylation (ICM; Figure 4A). DNA methylation profiles showed significant differences between sites and seasons (PERMANOVA, *p* < 0.05, Table S5), with seasonal variation accounting for 3.31% of total methylation profile variance, whereas site explained only 0.23%. This pattern appeared in both DAPC clustering (Figure 4C) and heatmap visualization (Figure S7A). DAPC analysis identified 47.97% (*n* = 130) of polymorphic MSL as most influential for discriminating methylation profiles across spatiotemporal gradients (Figure S6A,B).

**FIGURE 5:**
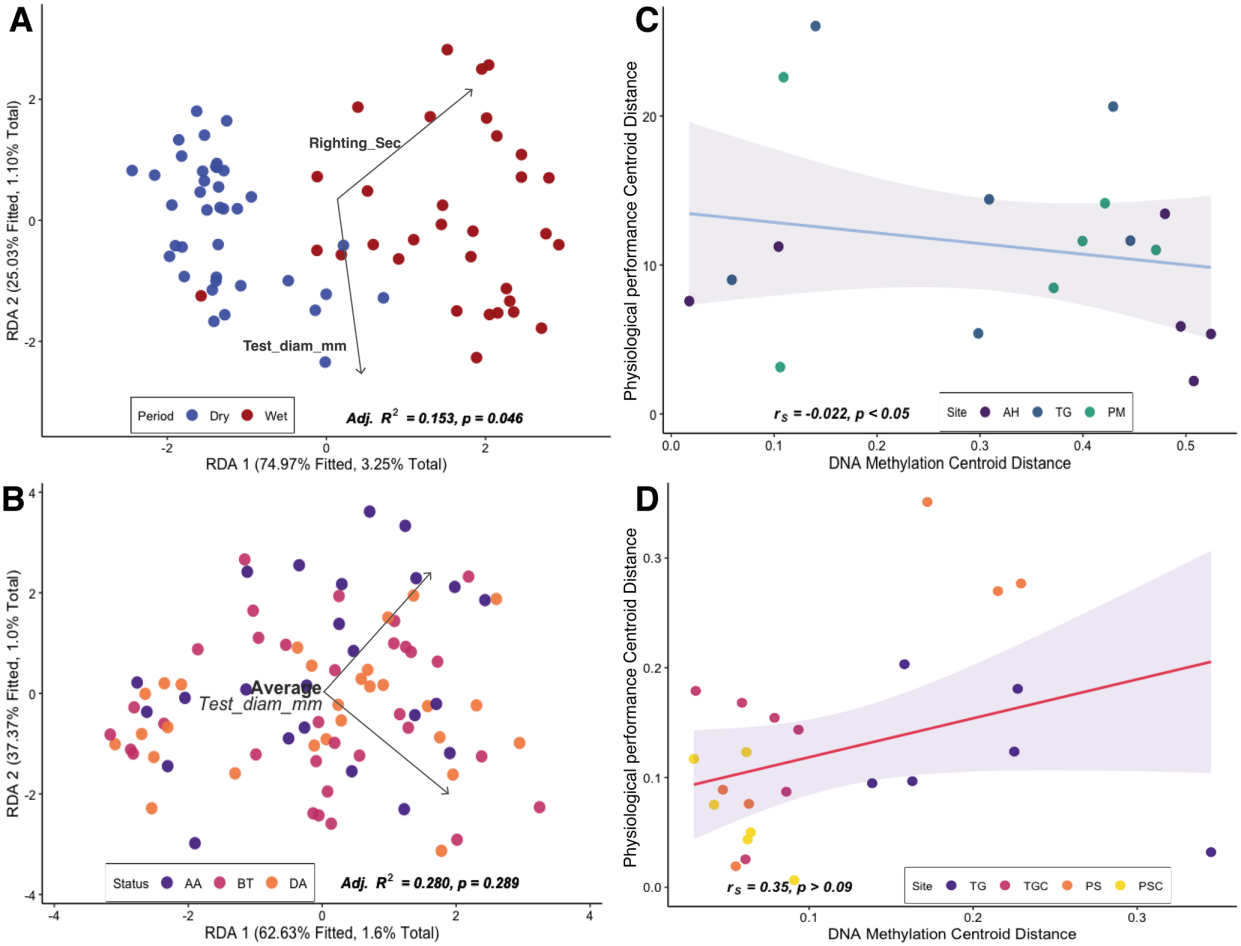
Relationship between physiological response (righting time) and epigenetic modifications in monitored (A–C) and transplanted (B–D) *D. antillarum* . (A, B) Distance‐based redundancy analysis (dbRDA) examining relationships between physiological (righting time) and morphological (test diameter) metrics and DNA methylation profiles of the most informative/susceptible loci identified through MSAP (Figure S6). (A) DNA methylation profiles per specimen are differentiated by season (blue: Dry season, January–April; red: Wet season, May–November), with spatial distribution reflecting the contribution of righting response to explaining DNA methylation variation across seasonal transitions. (B) specimen‐specific methylation profiles are differentiated by experimental phase (BT: Pre‐transplant, pink; DA: During acclimatization, yellow; AA: Post‐acclimatization, purple), demonstrating temporal epigenetic response progression. (C) Quantitative phenotypic‐epigenetic seasonal plasticity correlation. Euclidean distances between site combination centroids across seasonal timepoints map DNA methylation plasticity against physiological plasticity based on righting response. (D) Quantitative phenotypic‐epigenetic plasticity correlation in transplant response. Euclidean distances between experimental timepoint centroids map DNA methylation plasticity against physiological plasticity, with site‐specific chromatic differentiation. Physiological Performance Centroid Distance (y‐axis) corresponds to the physiological performance based on the Righting time measurements. Statistical relationships are quantified through Spearman's rank correlation coefficient (rS) with significance values (*p*) calculated via *cor.test* function. Linear relationships are visualized through regression lines with 95% confidence intervals (shaded regions). The most informative loci correspond to the loci with loading scores in the 90th percentile of the discriminant analysis, labeled and identified as the most influential loci, representing methylation‐susceptible sites with the strongest statistical associations between epigenetic state and experimental grouping (Figure S6).

DNA Methylation patterns in the most influential loci were significantly correlated with physiological measurements (dbRDA, *p* = 0.046), explaining 4.35% of DNA methylation variation. These loci represent DNA methylation‐susceptible sites exhibiting the strongest statistical associations with physiological traits, identified through multivariate analyses ranking loci by their epigenetic‐phenotypic correlation strength. The analysis of physiological metrics revealed that the righting response contributed significantly to the portion of DNA methylation variance explained (*p* = 0.028), while test diameter showed no significant contribution (*p* = 0.324; Figure 5A). Also, there was a significant relationship detected between the degree of seasonal phenotypic plasticity and epigenetic plasticity when comparing distances between centroids of sample sets from the same site across seasons (*p < 0.05*; Figure 5C).

### 3.4 Physiological and Epigenetic Shifts During Responses to Reciprocal Transplant

Environmental conditions during transplantation (March–June 2024) exhibited pronounced site‐specific heterogeneity. Seawater temperature profiles revealed systematic warming (Figure S2B,C). Salinity, sedimentation rate, and nutrient concentration remained consistent with the patterns observed in the monitoring period (Figures S2E–G), with lower values at PS than TG.

**FIGURE 6:**
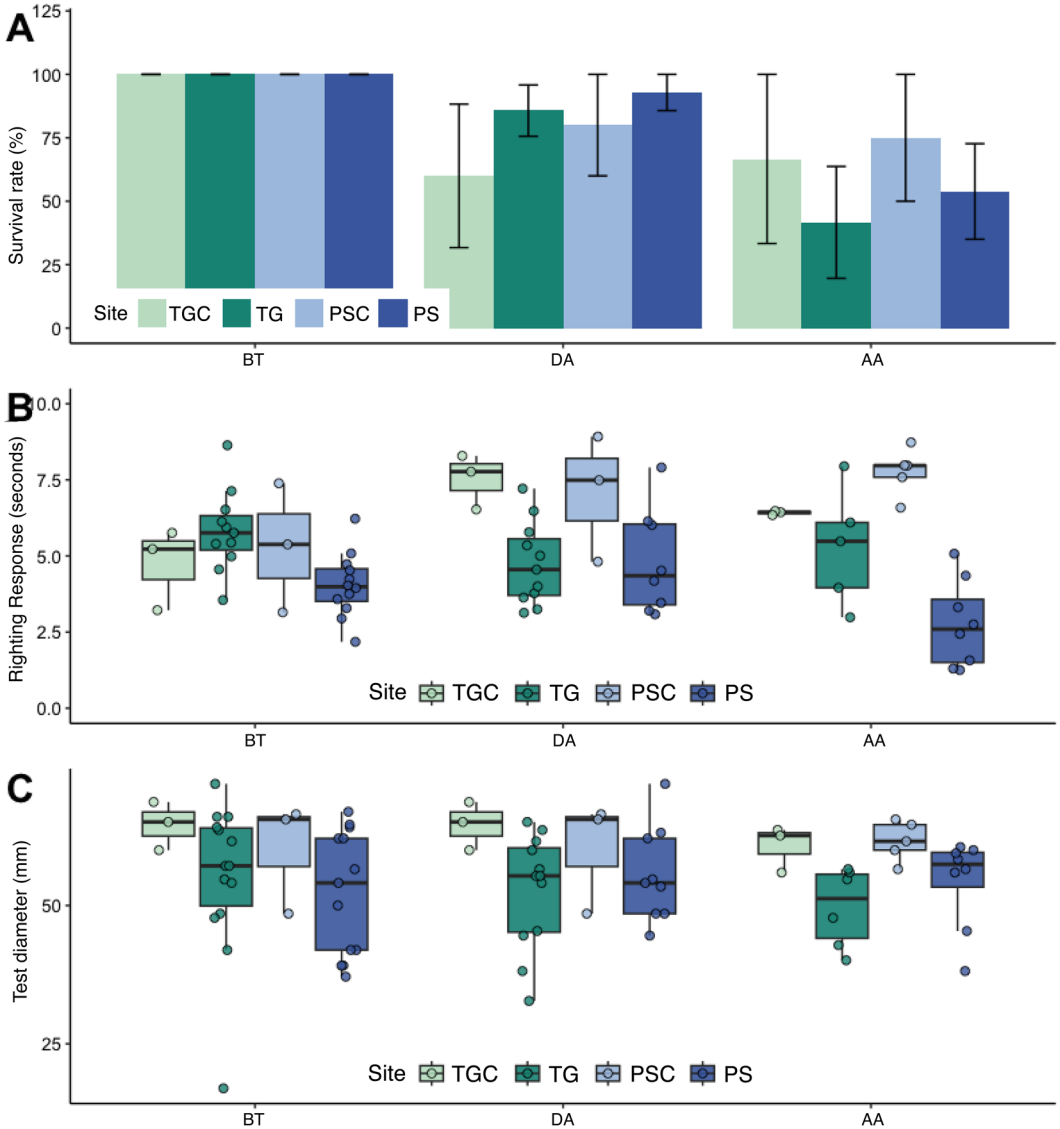
Survivorship, physiological performance (righting response), and morphological metrics (test diameter) during the reciprocal transplantation experiment. (A) Temporal analysis of survival rates across sites (PSC, Punta Soldado Control; PS, Transplanted to Punta Soldado; TGC, Tamarindo Grande Control; TG, Transplanted to Tamarindo Grande) and transplant experimental status category (AA, Post‐acclimatization/final experimental phase; BT, Pre‐transplant; DA, During acclimatization) in transplanted urchin populations. Error bars indicate ±1 SE. (B) Righting response capacity across experimental status. (C) Test diameter (mm) measurements across experimental status between sites. Boxplot elements show median (horizontal line), interquartile range (25th–75th percentiles), and whiskers extending to 1.5 × IQR. Individual data points are overlaid to show sample dispersion. (TG, PS, TGC, PSC as above.)

Twenty‐eight urchins were transplanted between TG and PS (fourteen per site), with ten additional urchins used as controls. Survival rates of experimental urchins differed among transplant status categories (BT, DA, AA; *χ* ^2^, *p* < 0.001). During the acclimatization period (DA), the population exhibited relatively high but variable survival, with mean rates ranging from approximately ~60% to ~90% depending on site and transplant status (Figure 6A). Following post‐acclimatization (AA), survival trajectories diverged markedly between controls and transplanted cohorts. Control populations (TGC, PSC) maintained survival rates of approximately ~66.6% and ~75%, respectively, while transplanted individuals experienced substantially elevated mortality, with survival declining to approximately ~41.6% at TG and ~53.8% at PS. Despite these pronounced temporal dynamics and the apparent survival differential between control and transplant groups, generalized linear modeling detected no statistically significant effects of destination site or experimental status on short‐term survival outcomes (GLM, site effect *p* = 0.797; status effect *p* = 0.170).

Physiological performance demonstrated more nuanced responses to transplantation. Righting time showed significant site‐dependent variation (ANOVA, *p* = 0.0003) and site‐status interactions (ANOVA, *p* < 0.001). In general, urchins at PS showed faster righting times than those at TG (Figure 6B). Control groups (TGC, PSC) exhibited significant differences from BT and AA groups, showing slower righting responses than the urchins transplanted. Notably, transplanted urchins reached righting times under nine seconds at AA, significantly faster than the earlier monitoring‐period individuals (Figure 6B, Figure S3).

Additionally, test diameter differed significantly between sites (ANOVA, *p = 0.004*), but not between transplant status categories (ANOVA, *p* > 0.05). Control groups were larger than transplanted urchins; BT and DA individuals were larger at TG, whereas at AA, the larger individuals were found at PS (Figure 6C). The correlation between test diameter and righting time persisted in the transplanted urchins (Spearman's *ρ* = 0.31, *p* < 0.01; Figure S4B), suggesting that biomechanical scaling relationships between body size and locomotory performance operate consistently across different environmental contexts and experimental conditions.

Multivariate analysis revealed significant physiological performance differences between sites (PERMANOVA, *p* = 0.001) and transplant categories (PERMANOVA, *p* = 0.003), with site and status together explaining 13% (site = 0.58% and status = 12.44%) of physiological performance variance (RDA, *p* = 0.005; Figure S5C, Tables S3 and S4).

Epigenetically, transplanted individuals also exhibited significant changes. MSAP analysis identified 111 MSL among the transplant samples; 108 (97%) were polymorphic. Shannon's diversity index (SDI = 0.456 ± 0.154, Mean ± SD) revealed substantial epigenetic variation. The methylation state distribution mirrored our monitoring patterns, with predominant hypermethylation (HPM; Figure 4B). Transplant status and site significantly influenced methylation profiles (RDA, *p* = 0.001 for both), with transplant status explaining substantially more variance (12.44%) than site (0.58%). DAPC clustering confirmed the primary differentiation by transplant status and secondary by site (Figure 4D). This pattern was validated by heatmap visualization (Figure S7B). Nearly half (48.39%, *n* = 124) of polymorphic MSL were identified as most influential in differentiating transplant categories (Figure S6C,D).

However, no significant correlation appeared between DNA methylation patterns and physiological performance (dbRDA, *p* = 0.289), with the model explaining only 2.63% of DNA methylation variance (Figure 5B). Furthermore, no significant relationship emerged between the magnitude of phenotypic and epigenetic plasticity (Figure 5D *p* > 0.05).

## 4 Discussion

### 4.1 Environmental Influences on *D. antillarum* Population and Physiological Performance

Pronounced seasonal and site‐specific variations in seawater temperature, salinity, sedimentation, and nutrients established environmental gradients that correlate with distinct physiological responses in the *D. antillarum* populations. The observed seawater temperature range (GLM, *p* < 0.01), seasonal salinity fluctuations (GLM, *p* < 0.001), and sedimentation regimens (GLM, *p* < 0.001) reflect the complex hydrographic regimes characteristic of tropical shallow‐water ecosystems, where seasonal precipitation patterns, terrestrial runoff dynamics, and localized oceanographic circulation interact to create site‐specific environmental signatures (Bowden et al. 2021; Jury 2022; Thouvenin‐Masson et al. 2024).

*Diadema antillarum* densities were inversely correlated with sedimentation, which affects the urchins' feeding efficiency and establishment under elevated sediment loading (Chiarore et al. 2020; Kobelt et al. 2020). The righting response, a proxy for neuromuscular function (Ebert 1997, 2019; Lawrence and Cowell 1996; McDonald et al. 2022), varied markedly among sites. Urchins at AH displayed superior performance than their TG and PM counterparts; this pattern is consistent with favorable environmental conditions—such as reduced salinity fluctuations, seawater temperatures within the species' optimal range (approx. 24°C–29°C; Hassan et al. 2022; Sherman 2015; Wijers et al. 2023), enhanced circulation, suitable substrate conditions, and minimal nutrient inputs—that can modulate physiological and behavioral functions (Bodmer et al. 2017; Hassan et al. 2022; Sherman 2015). In contrast, elevated sedimentation likely imposed greater stress on echinoids through multiple mechanisms, including reduction in settlement success, impaired respiratory efficiency, reduced feeding efficiency, and increased metabolic costs associated with sediment removal (Chiarore et al. 2020; Glockner‐Fagetti and Phillips 2019; Kobelt 2019). Urchins at TG had larger test diameters, but slower righting times compared to individuals at AH and PM (Figure 3A,B). This pattern is consistent with well‐established biomechanical constraints associated with larger body size (greater body mass and longer spines) that may increase righting time (Chaar et al. 2024; Challener and McClintock 2017; Hagen 2020). The positive correlation between test diameter and righting time (Spearman's *ρ* = 0.30, *p* < 0.001; Figure S4A) is therefore most parsimoniously attributed to these physical constraints rather than necessarily indicating compromised physiological condition in larger urchins.

The observed seasonal variation also influenced the physiological plasticity of *D. antillarum* , with improved righting responses in the dry season compared to the wet season, paralleling lower sedimentation inputs. Salinity and temperature fluctuations combined with sedimentation‐induced stress (particularly during the wet season) likely exacerbate physiological performance as indicated by reduced righting response (Barrett et al. 2024; Bodmer et al. 2017; Himmelman et al. 1984; Irlandi et al. 1997; Okamoto et al. 2020).

### 4.2 Environmental Drivers of Epigenetic Plasticity in *D. antillarum*

We identified 294 methylation‐susceptible loci (MSL), with 98% polymorphism, indicating substantial inter‐individual epigenetic variability consistent with patterns across marine taxa (Liew et al. 2018; Putnam et al. 2016). The predominance of HPM supports the notion that extensive DNA methylation may promote genomic stability (Dang et al. 2023; Yang et al. 2020). While global DNA methylation patterns remained relatively consistent spatially, multivariate analyses revealed significant temporal shifts. Seasonal variation explained roughly 3.31% of epigenetic variance, versus 0.23% for spatial differences, suggesting that dynamic environmental fluctuations (seawater temperature regimes, salinity oscillations, nutrient and sedimentation pulses) exert more influence on DNA methylation than static site differences (Dimond and Roberts 2016; Hackerott et al. 2023; Liu et al. 2023). The discriminant analysis of principal components (Figure 4C) corroborated this, showing clear seasonal segregation. These seasonal DNA methylation patterns likely reflect acclimatory epigenetic responses in *D. antillarum* as a flexible response mechanism to changing environments.

Distance‐based redundancy analysis of the monitoring data showed a significant relationship between DNA methylation patterns and righting response, suggesting that this epigenetic modulation may be associated with physiological pathways essential for stress resilience, such as those involved in energy metabolism, cytoskeletal dynamics, or locomotion, thereby affecting righting ability (Bogan et al. 2023; Strader et al. 2020). Conversely, no significant association emerged between test diameter and DNA methylation profiles (dbRDA, *p* = 0.324; Figure 5A). Test diameter represents a cumulative morphometric trait integrating multiple biological processes, including age‐related accumulation of growth, genetic determination of growth rates, resource availability, and historical environmental conditions over an individual's lifespan (Ebert 2019; Levitan 1988, 1989), rather than epigenetic responses to environmental shifts (Liu et al. 2023; Strader et al. 2020).

### 4.3 Environmental Effects of Reciprocal Transplants on Physiological Performance and Acclimatization Capacity

The reciprocal transplant experiment revealed a decoupling between individual‐level physiological plasticity and population‐level survival: surviving transplanted urchins exhibited rapid recovery of neuromuscular function, whereas overall survival declined markedly, reaching approximately 41.6% at TG and 53.8% at PS by the post‐acclimatization period (AA; Figure 6A).

Despite pronounced environmental heterogeneity between TG and PS, surviving transplanted urchins showed better physiological performance related to controls during the post‐acclimatization period AA, especially at PS (Figure 6B). All urchins flipped consistently under ≈9 s, much faster than those observed during the 11‐month monitoring period (Figure 3A). This improved performance likely reflects both the smaller body size of experimental urchins (Figures 3C, 6C; Chaar et al. 2024; Challener and McClintock 2017; Hagen 2020) and the population recovery following the 2022 mortality event. Our results likely indicate that the righting response appears to be an indicator for evaluating the sea urchin health and condition (Chaar et al. 2024). Furthermore, these results may indicate that *D. antillarum* exhibits short‐term physiological acclimatization, maintaining functional stability under variable environmental conditions, suggesting DNA methylation can acts as an environmentally responsive epigenetic regulatory mechanism priming the organism for prolonged stress as shown in other invertebrates (Eirin‐Lopez and Putnam 2019; Rodríguez‐Casariego et al. 2020; Shi and Li 2024; Strader et al. 2020).

However, survival analyses revealed substantial mortality across all transplanted groups, with transplanted urchins experiencing absolute losses than controls (~50% mortality; Figure 6A). Survival declined similarly at both sites during the post‐acclimatization phase, with no significant effect of destination or status (GLM, site effect *p* = 0.797; status effect *p* = 0.170). This absence of site‐specific or transplant‐specific survival differences, coupled with procedural stress (i.e., transportation, handling, caging, tissue sampling) appeared to be a primary mortality driver rather than environmental incompatibility between origin and destination habitats. This pattern implies that procedural stress itself imposes greater physiological costs than environmental differences. When these disturbances are minimized, *D. antillarum* appears capable of tolerating diverse conditions given adequate habitat structure and food availability (Bodmer et al. 2021).

DNA methylation analyses paralleled these physiological performance findings. Transplant status explained a greater fraction of DNA methylation variance (12.44%) than site (0.58%), reflecting that temporal status of the transplantation process exerted stronger epigenetic effects rather than habitat‐specific environmental differences. Thus, DNA methylation alterations in transplanted *D. antillarum* likely reflect combined handling stress and environmental effects, with epigenetic mechanisms modulating metabolic and behavioral functions (Bogan et al. 2023; Liu et al. 2023).

The predominance of hypermethylation (HPM; Figure 4B) across all transplant categories mirrors patterns observed during the monitoring period, suggesting a conserved baseline DNA methylation architecture. However, despite significant epigenetic shifts associated with transplantation, we found no significant correlation between DNA methylation and physiological performance in transplanted individuals (dbRDA, *p =* 0.289; Figure 5B), suggesting *D. antillarum* acclimatization capacity likely involves additional regulatory mechanisms, beyond DNA methylation, including post‐transcriptional regulation, protein modification, or metabolic reprogramming as observed in other marine invertebrates (Bogan et al. 2023; Hofmann 2017; Strader et al. 2024). Transplanted urchins often retained normal function in novel habitats, demonstrating pronounced plasticity (Pilnick et al. 2023; Strader et al. 2020), although the precise molecular mechanisms remain unresolved. However, recovery in survivors does not guarantee demographic recovery if many individuals die (Pilnick et al. 2023; Strader et al. 2020). Translating this individual‐level plasticity into demographic success for restoration programs requires a substantial reduction of transplantation‐associated procedural mortality. In this experiment, rapid physiological recovery in surviving urchins occurred alongside ~50% mortality, highlighting that apparent acclimatization must be interpreted considering cohort‐level losses.

### 4.4 Implications for *D. antillarum* Conservation

Our analyses underscore the significant contribution of DNA methylation to environmental responses in *D. antillarum* , supporting the integration of epigenetic characterization into restoration efforts. We identified 294 methylation‐susceptible loci (98% polymorphic) in the monitored urchins significantly associated with righting response performance, suggesting DNA methylation mediates physiological acclimatization in this species (Bogan et al. 2023; Strader et al. 2020). However, this plasticity comes with measurable physiological costs, evidenced by negative correlations between urchin density and sedimentation rates, alongside compromised righting responses at high‐stress sites (e.g., densities at TG).

Our results have practical implications for *D. antillarum* restoration strategies. Both field surveys and transplant experiments show that elevated sedimentation and nutrient imbalance were correlated with lower urchin density and slower righting responses. Sites exceeding ~30 mg·cm^−2^·day^−1^ sediment deposition or N:P ratios > 22:1 impose clear physiological stress. Reciprocal transplantation suggests acclimatization capacity to elevated sedimentation and nutrient loads, with transplant status explaining 12.44% of DNA methylation variance (Figure S5C). Nevertheless, transplantation to severely stressed environments (sedimentation > 30 mg·cm^−2^·day^−1^, N:*p* > 22:1) required caution, ideally preceded by habitat remediation and preconditioning protocols to minimize procedural mortality.

The shown epigenetic plasticity suggests potential resilience, reinforcing the value of *D. antillarum* for reestablishing herbivory and supporting coral restoration (Cano et al. 2021; Hylkema et al. 2022; Latijnhouwers et al. 2024; Mumby and Steneck 2008; Williams 2022). If this epigenetic modification persists, transgenerational heritable modifications may enhance offspring's fitness under similar conditions (Eirin‐Lopez and Putnam 2019; Putnam and Gates 2015). While such epigenetic markers may eventually inform *D. antillarum* resilience selection or guide preconditioning strategies—similar to successful coral restoration approaches (Martell et al. 2025; Putnam and Gates 2015)—conservation planning should presently prioritize empirically validated site conditions and sea urchin performance metrics rather than untested molecular markers.

## 5 Conclusions

Our study addresses a critical knowledge gap in Caribbean reef restoration ecology by establishing quantitative linkages between habitat quality, epigenetic regulation, and physiological performance in *D. antillarum*. Our results suggest that environmental gradients in seawater temperature, salinity, sedimentation, and nutrient loading influence righting performance and survival, while DNA methylation provides a dynamic regulatory mechanism modulating these responses. Reciprocal transplant experiments revealed that procedural stress outweighed environmental differences in determining short‐term mortality, emphasizing that procedural refinement is essential for successful restoration practices.

In addition, this work provides the first comprehensive characterization of DNA methylation in *D. antillarum* , providing molecular baselines with potential applications for species recovery and restoration programs. Since the temporal stability of those epigenetic modifications are critical for understanding the evolutionary significance of these response mechanisms, future research should evaluate the temporal stability, potential transgenerational inheritance patterns, and functional genomic consequences of DNA methylation changes, facilitating the development of molecular biomarker useful for restoration. Overall, strengthening the links between phenotypic plasticity and epigenetic dynamics will be critical to better inform evidence‐based restoration strategies, improving the efficacy of herbivore‐based reef restoration strategies in climate‐driven marine environments.

## Author Contributions

**Ibis T. Lopez‐Jimenez:** conceptualization (equal), data curation (equal), formal analysis (equal), investigation (equal), methodology (equal), visualization (equal), writing – original draft (equal), writing – review and editing (equal). **Alex E. Mercado‐Molina:** conceptualization (equal), methodology (equal), resources (equal), writing – review and editing (equal). **Juliet M. Wong:** conceptualization (equal), data curation (equal), formal analysis (equal), funding acquisition (equal), investigation (equal), methodology (equal), project administration (equal), resources (equal), software (equal), supervision (equal), validation (equal), visualization (equal), writing – original draft (equal), writing – review and editing (equal). **Jose Eirin‐Lopez:** conceptualization (equal), data curation (equal), formal analysis (equal), funding acquisition (equal), investigation (equal), methodology (equal), project administration (equal), resources (equal), supervision (equal), validation (equal), visualization (equal), writing – original draft (equal), writing – review and editing (equal).

## Funding

This work was supported by Puerto Rico Sea Grant, University of Puerto Rico (NA22OAR4170097).

## Conflicts of Interest

The authors declare no conflicts of interest.

## Supporting information


**Appendix S1:** Supporting Information.

**Appendix S2:** Supporting Information.

## Data Availability

Datasets utilized in the study, which characterize environmental conditions, *D. antillarum* physiology, and MSAP loci, along with the associated metadata and data analysis R Script can be found in the Zenodo repository (https://zenodo.org/records/17443949) DOI: 10.5281/zenodo.17443949.
