# Supplementary material for: Physiological and Epigenetic Responses of the Long‐Spined Sea Urchin Diadema antillarum Across a Spatiotemporal Gradient
Source: Ecol Evol. 2026 Jan 12;16(1):e72915. doi: 10.1002/ece3.72915 (PMC12793898; doi:10.1002/ece3.72915)
Supplement: Supplementary file 1 — Appendix S1: Supporting Information. [file ECE3-16-e72915-s001.docx]

**Supplemental Data Tables**

Table S1……………………………………………………………………………….4

Table S2……………………………………………………………………………….5

Table S3……………………………………………………………………………….6

Table S4……………………………………………………………………………….7

Table S5……………………………………………………………………………….9

Table S6……………………………………………………………………………….10

Table S7……………………………………………………………………………….12

Table S8……………………………………………………………………………….13

**Supplemental Data Figures**

Figure S1………………………………………………………………………………21

**Table S1. Total count of *Diadema antillarum* individuals surveyed across four study sites during monitoring sampling and transplant experiment using six 10 × 2 m belt transects per site.**

| **A) Surveys during the monitoring period** | | | | | | | |
| --- | --- | --- | --- | --- | --- | --- | --- |
| Season | **DRY** | | | **WET** | | |  |
|  | **S** | **M** | **L** | **S** | **M** | **L** | **TOTAL** |
| **TG** | *6* | *4* | *14* | *7* | *17* | *18* | ***66*** |
| **PM** | *9* | *7* | *3* | *22* | *34* | *10* | ***85*** |
| **AH** | *19* | *22* | *9* | *31* | *40* | *31* | ***152*** |
| **PS** | *0* | *0* | *0* | *2* | *2* | *1* | ***5*** |
| **TOTAL** | ***54*** | ***78*** | ***66*** | ***77*** | ***112*** | ***73*** | **308** |
|  | | | | | | | |
| **B) Surveys during the transplant period** | | | | | | | |
| Season | **DRY** | | | **WET** | | |  |
|  | **S** | **M** | **L** | **S** | **M** | **L** | **TOTAL** |
| **TG** | *6* | *17* | *12* | *7* | *7* | *4* | ***119*** |
| **PS** | *9* | *16* | *18* | *8* | *12* | *9* | ***77*** |
| **TOTAL** | ***54*** | ***78*** | ***66*** | ***77*** | ***112*** | ***73*** |  |
|  |  |  |  |  |  |  |  |
| **TOTAL over full experiment:** | | | | | | | **460** |

**Table S2. Mean and Standard deviation of *Diadema antillarum* density and physiological metrics across sites for both, monitored and transplanted sea urchins.** Analyzed metrics include: (A) Density (individuals/m^2^), (B) Righting response (seconds) and (C) Test diameter (mm).

| 1. **Density (individuals/m^2^)** | | | | | | |  |
| --- | --- | --- | --- | --- | --- | --- | --- |
|  | **General** | | **Dry Season** | | **Wet Season** | | |
|  | **Mean** | **SD** | **Mean** | **SD** | **Mean** | **SD** | |
| Tamarindo Grande (TG) | *0.058* | *0.078* | *0.089* | *0.053* | *0.040* | *0.023* | |
| Punta Melones (PM) | *0.117* | *0.058* | *0.127* | *0.084* | *0.110* | *0.046* | |
| La Ahogada (AH) | *0.182* | *0.051* | *0.208* | *0.058* | *0.171* | *0.050* | |
| Punta Soldado (PS) | *0.090* | *0.043* | *0.122* | *0.107* | *0.071* | *0.057* | |
|  | | | | | | |  |
| 1. **Righting Response (Seconds)** | | | | | | |  |
| **B1. Urchins Monitored** | | | | | | |  |
|  | **General** | | **Dry Season** | | **Wet Season** | | |
|  | **Mean** | **SD** | **Mean** | **SD** | **Mean** | **SD** | |
| Tamarindo Grande (TG) | *12.470* | *5.774* | *12.311* | *2.870* | *12.568* | *7.051* | |
| Punta Melones (PM) | *8.514* | *2.774* | *10.039* | *2.499* | *6.698* | *1.857* | |
| La Ahogada (AH) | *8.739* | *1.434* | *9.729* | *4.468* | *9.178* | *5.426* | |
| **B2. Urchins Transplanted** | | | | | | |  |
|  | **General** | | **Dry Season** | | **Wet Season** | | |
|  | **Mean** | **SD** | **Mean** | **SD** | **Mean** | **SD** | |
| Tamarindo Grande (TG) | *8.855* | *6.847* | *7.923* | *4.372* | *10.485* | *10.019* | |
| Punta Soldado (PS) | *3.940* | *2.018* | *4.616* | *2.036* | *2.757* | *1.419* | |
| Tamarindo Grande, control (TGC) | *6.227* | *1.461* | *6.132* | *1.839* | *6.418* | *0.068* | |
| Punta Soldado, control (PSC) | *6.908* | *1.778* | *6.190* | *2.115* | *7.770* | *0.780* | |
|  | | | | | | |  |
| 1. **Test Diameter (mm)** | | | | | | |  |
| **C1. Urchins Monitored** | | | | | | |  |
|  | **General** | | **Dry Season** | | **Wet Season** | | |
|  | **Mean** | **SD** | **Mean** | **SD** | **Mean** | **SD** | |
| Tamarindo Grande (TG) | *64.238* | *19.969* | *63.875* | *17.262* | *64.461* | *21.795* | |
| Punta Melones (PM) | *48.739* | *13.433* | *49.000* | *12.734* | *48.428* | *14.534* | |
| La Ahogada (AH) | *46.596* | *9.472* | *45.965* | *9.443* | *47.391* | *9.661* | |
| **C2. Urchins Transplanted** | | | | | | |  |
|  | **General** | | **Dry Season** | | **Wet Season** | | |
|  | **Mean** | **SD** | **Mean** | **SD** | **Mean** | **SD** | |
| Tamarindo Grande (TG) | *55.909* | *17.168* | *61.000* | *18.489* | *47.000* | *10.337* | |
| Punta Soldado (PS) | *55.954* | *14.443* | *55.071* | *16.288* | *57.500* | *11.351* | |
| Tamarindo Grande, control (TGC) | *72.777* | *8.348* | *75.33* | *8.066* | *67.666* | *7.571* | |
| Punta Soldado, control (PSC) | *68.636* | *11.826* | *68.000* | *15.517* | *69.400* | *6.877* | |

**Table S3: Generalized linear model results for environmental parameters.** Effects of site, season, and their interaction on: (A) Temperature (°C), (B) Salinity (ppt), (C) Sedimentation rate (g/cm²/day), and (D) Nutrient concentrations (µM). Models include the daily average and standard deviation of temperature and salinity, and nutrient concentrations (total nitrogen, total phosphorus, and N:P ratio) measured every six weeks. Model distributions and p-values (based on likelihood ratio chi-square tests) are reported. Significant values (α = 0.05) are bolded.

| **A.** | **Temperature (°C) Average** | | **Temperature (°C) Std. Deviation** | |  |
| --- | --- | --- | --- | --- | --- |
| Distribution | Inverse Gaussian | | Inverse Gaussian | |  |
| Site | ***< 0.001*** | | ***< 0.001*** | |  |
| Season | ***< 0.001*** | | ***< 0.001*** | |  |
| Site x Season | ***< 0.001*** | | ***< 0.001*** | |  |
|  | | | | |  |
| **B.** | **Salinity (ppt) Average** | | **Salinity (ppt) Std. Deviation** | |  |
| Distribution | **Gaussian** | | Gaussian | |  |
| Site | ***< 0.001*** | | ***< 0.001*** | |  |
| Season | ***< 0.001*** | | ***< 0.001*** | |  |
| Site x Season | ***< 0.001*** | | ***< 0.001*** | |  |
|  | | | | |  |
| **C.** | **Sedimentation rate (g/cm2/day)** | | **Sedimentation rate (g/cm2/day)** | |  |
| Distribution | Inverse Gaussian | | Gamma | |  |
| Site | ***< 0.001*** (3.72x10^-08^) | | ***< 0.001*** (3.58x10^-08^) | |  |
| Season | 0.091 | | 0.089 | |  |
| Site x Season | *0.157* | | *0.411* | |  |
|  | | | | |  |
| **D.** | **Nitrogen (µM)** | **Phosphorus (µM)** | | **N:P** | |
| Distribution | Gaussian | Gamma | | Inverse Gaussian | |
| Site | *0.339* | *0.260* | | *0.098* | |
| Season | ***0.0057*** | *0.609* | | ***0.0018*** | |
| Site x Season | *0.410* | *0.250* | | *0.225* | |

**Table S4. Pairwise comparisons of temperature and salinity by season and site.** A1 and B1: Pairwise comparisons of daily average temperature and salinity across seasons. A2 and B2: Comparisons of daily standard deviations across sites. Upper diagonal shows comparisons using estimated marginal means (*emmeans* function). Significant *p-values* (α = 0.05) are in bold.

| **A. Temperature (ºC)** | | | | |  | |  |
| --- | --- | --- | --- | --- | --- | --- | --- |
| **A1. Season** |  | | | |  | |  |
|  | **Dry** | | **Wet** | |  |  |  |
| **Dry** | *NA* | | ***< 0.001*** | |  |  |  |
| **Wet** | *NA* | | *NA* | |  |  |  |
|  | | | | |  |  |  |
| **A2. Site Comparison** | | | | | |  | |
|  | **AH** | **PS** | | **PM** | | **TG** | |
| **AH** |  | *0.999* | | *0.873* | | ***0.010*** | |
| **PS** | *NA* |  | | *0.809* | | ***0.013*** | |
| **PM** | *NA* | *NA* | |  | | ***0.001*** | |
| **TG** | *NA* | *NA* | | *NA* | |  | |
| **B. Salinity (ppt)** |  | | | |  | |  |
| **B1. Season** |  | | | |  | |  |
|  | **Dry** | | **Wet** | |  |  |  |
| **Dry** | *NA* | | ***<0.0001*** | |  |  |  |
| **Wet** | *NA* | | *NA* | |  |  |  |
|  | | | | |  |  |  |
| **B2. Site Comparison** | | | | | |  | |
|  | **AH** | **PS** | | **PM** | | **TG** | |
| **AH** |  | ***<0.0001*** | | ***<0.0001*** | | ***<0.0001*** | |
| **PS** | *NA* |  | | ***<0.0001*** | | ***<0.0001*** | |
| **PM** | *NA* | *NA* | | *NA* | | ***<0.0001*** | |
| **TG** | *NA* | *NA* | | *NA* | |  | |

**Table S5. Analysis of variance (ANOVA) results of urchin metrics across sites and seasons.** Analyzed variables include: (A) Density, (B) Righting response (seconds), and (C) Size surveyed and Test diameter (mm), (D) Survival rate. Significant p-values (α = 0.05) are in bold.

| 1. **Density** |  | | | | | | | | | | | | |
| --- | --- | --- | --- | --- | --- | --- | --- | --- | --- | --- | --- | --- | --- |
|  | Df | | Sum Sq. | | Mean Sq. | | F-value | | | Pr (>F) | | | |
| Site | *3* | | *0.1107* | | *0.03693* | | *25.735* | | | ***0.001****** | | | |
| Season | *1* | | *0.0003* | | *0.0139* | | *0.212* | | | *0.057** | | | |
| Site x Season | *3* | | *0.0045* | | *0.00150* | | *1.047* | | | *0.394* | | | |
|  | | | | | | | | | | | | | |
| 1. **Righting Response (Seconds)** | | | | | | | | | | | | |  |
| **B1. Urchins Monitored** | | | | | | | | | | | | |  |
|  | | Df | | Sum Sq. | | Mean Sq. | | F-value | | | | Pr (>F) |  |
| Site | | *2* | | *371.2* | | *185.59* | | *8.905* | | | | ***0.0002****** |  |
| Season | | *1* | | *53.3* | | *53.30* | | *2.557* | | | | *0.1121* |  |
| Site x Season | | *2* | | *78.7* | | *39.33* | | *1.887* | | | | *0.1555* |  |
| **B2. Urchins Transplanted** | | | | | | | | | | | | |  |
|  | | Df | | Sum Sq. | | Mean Sq. | | | F-value | | Pr (>F) | |  |
| Site | | *3* | | *2.569* | | *0.856* | | | *5.006* | | ***0.0031 ***** | |  |
| Status | | *2* | | *0.155* | | *0.077* | | | *0.455* | | *0.6361* | |  |
| Site x Status | | *6* | | *3.061* | | *0.510* | | | *2.892* | | ***0.0113 **** | |  |
|  | | | | | | | | | | | | |  |
| 1. **Size monitoring and Test Diameter (mm)** | | | | | | | | | | | | |  |
| **C1. Size of urchins surveyed on transects** | | | | | | | | | | | | |  |
|  | | Df | | Sum Sq. | | Mean Sq. | | F-value | | | | Pr (>F) |  |
| Site | | *3* | | *430* | | *157.67* | | *3.780* | | | | ***0.0318**** |  |
| Season | | *1* | | *170.7* | | *170.67* | | *4.092* | | | | *0.0601* |  |
| Site x Season | | *3* | | *360.3* | | *120.11* | | *2.880* | | | | *0.0684* |  |
| **C2. Urchins Monitored** | | | | | | | | | | | | |  |
|  | | Df | | Sum Sq. | | Mean Sq. | | | F-value | | Pr (>F) | |  |
| Site | | *2* | | *8249* | | *4124* | | | *19.048* | | ***5.24x10^-8^ ****** | |  |
| Season | | *1* | | *9* | | *9* | | | *0.042* | | *0.838* | |  |
| Site x Season | | *2* | | *24* | | *12* | | | *0.056* | | *0.946* | |  |
| **C3. Urchins Transplanted** | | | | | | | | | | | | |  |
|  | | Df | | Sum Sq. | | Mean Sq. | | | F-value | | Pr (>F) | |  |
| Site | | *3* | | *1.229* | | *0.409* | | | *4.757* | | ***0.0042 ***** | |  |
| Status | | *2* | | *0.097* | | *0.048* | | | *0.564* | | *0.5711* | |  |
| Site x Status | | *6* | | *0.324* | | *0.053* | | | *0.626* | | *0.7087* | |  |
|  | | | | | | | | | | | | |  |
| 1. **Survival rate transplanted urchin** | | | | | | | | | | | | |  |
|  | | Df | | Sum Sq. | | Mean Sq. | | F-value | | | | Pr (>F) |  |
| Site | | *3* | | *1188* | | *396.0* | | *0.713* | | | | *0.797* |  |
| Status | | *2* | | *5811* | | *2905.3* | | *5.230* | | | | *0.170* |  |
| Site x Status | | *6* | | *3371* | | *561.9* | | *1.011* | | | | *0.970* |  |

**Table S6. Multivariate analysis of variance in urchin physiological performance (Righting time + Test diameter).** *p-values* are displayed for PERMANOVA and PERMDISP and *p-values* less than a = 0.05 are in bold. (A) Monitoring period. (B) Transplant experiment.

| **A. Monitored *D. antillarum* (11-month period)** | | |
| --- | --- | --- |
| **A1.** | **PERMANOVA** | **PERMDISP** |
| Site | ***0.001*** | 0.339 |
| Season | ***0.002*** | 0.130 |
| Site x Season | ***0.008*** | NA |
|  | | |
| **A2.** | **PERMANOVA** | **PERMDISP** |
| Site | ***0.001*** | 0.021 |
| Status | ***0.003*** | < 0.001 |
| Site x Status | ***0.023*** | NA |
|  | | |
| **B. Transplanted D. antillarum (3-month experiment period)** | | |
| **A.** | **PERMANOVA** | **PERMDISP** |
| Site | ***0.001*** | 0.326 |
| Season | ***0.001*** | 3.503x10^-8^ |
| Site x Season | ***0.001*** | NA |
|  | | |
| **B.** | **PERMANOVA** | **PERMDISP** |
| Site | ***0.001*** | < 0.001 |
| Season | ***0.001*** | 0.691 |
| Site x Season | ***0.001*** | NA |

**Table S7. Linear model results for *D. antillarum* physiological performance (Righting time + Test diameter).** (A) Monitored individuals; (B) Transplanted individuals (B1 = by month, B2 = by transplant status). Site, season, and their interaction were included as predictors. Models report *p-values* and effect sizes (η²) using *anova* and *eta_squared*. Significant values (α = 0.05) are bolded.

| **A.** | | **Righting response (seconds)** | | | | **Test diameter (mm)** | | | |  |
| --- | --- | --- | --- | --- | --- | --- | --- | --- | --- | --- |
|  |  | *p-value* | | η^2^ | | *p-value* | | η^2^ | |  |
| Site | | ***0.0078*** | | *0.096* | | ***0.0007*** | | *0.1338* | |  |
| Season | | ***0.0089*** | | *0.074* | | ***0.0010*** | | *0.1856* | |  |
| Site x Season | | *0.3777* | | ***0.010*** | | *0.6450* | | *0.0364* | |  |
|  | | | | | | | | | |  |
| **B.1** | **Righting response (seconds)** | | | | **Test diameter (mm)** | | | |  |  |
|  | *p-value* | | η^2^ | | *p-value* | | η^2^ | |  |  |
| Site | ***0.0018*** | | *0.135* | | ***0.0050*** | | *0.148* | |  |  |
| Month | *0.7038* | | *0.011* | | *0.6022* | | *0.019* | |  |  |
| Site x Month | ***0.0016*** | | *0.250* | | *0.8379* | | *0.052* | |  |  |
|  | | | | | | | | |  |  |
| **B.2** | | | **Righting response (seconds)** | | | | **Test diameter (mm)** | | | |
|  |  |  | *p-value* | | η^2^ | | *p-value* | | η^2^ | |
| Site | | | ***0.0031*** | | *0.135* | | ***0.0042*** | | *0.148* | |
| Status | | | *0.6361* | | *0.008* | | *0.5711* | | *0.011* | |
| Site x Status | | | ***0.0113*** | | *0.161* | | *0.7087* | | *0.039* | |

**Table S8. DNA methylation profiles of *D. antillarum*.** Percentage of target sequences methylated per site.

| **A. Urchins Monitored,** | |  | |  | |  | |
| --- | --- | --- | --- | --- | --- | --- | --- |
| Band pattern (target state) | | **AH** | | **PM** | | **TG** | |
| HPA+/MSP+ (Unmethylated) – **NMT** | | 16.18 | | 16.28 | | 13.68 | |
| HPA + /MSP-(Hemimethylated) – **HMM** | | 18.82 | | 19.86 | | 18.08 | |
| HPA-/MSP + (Internal C methylation) – **ICM** | | 16.27 | | 17.93 | | 18.61 | |
| HPA-/MSP-(Hypermethylation) – **HPM** | | 48.72 | | 45.93 | | 49.62 | |
| Primer C1: 0, C2: 11, C3: 56, C4: 227 | |  | |  | |  | |
|  | | | | | | | |
| **B1. Urchins Transplanted,** | | | | | | | |
| Band pattern (target state) | **TG** | | **PS** | | **TGC** | | **PSC** |
| HPA+/MSP+ (Unmethylated) – **NMT** | 12.01 | | 14.15 | | 0.40 | | 2.70 |
| HPA + /MSP-(Hemimethylated) – **HMM** | 23.29 | | 22.34 | | 0.92 | | 9.58 |
| HPA-/MSP + (Internal C methylation) – **ICM** | 15.70 | | 17.30 | | 11.31 | | 8.60 |
| HPA-/MSP-(Hypermethylation) – **HPM** | 49.00 | | 46.21 | | 75.47 | | 79.11 |
| Primer C1: 6, C2: 45, C3: 58, C4: 0 |  | |  | |  | |  |

**Supplemental Data Figures and Captions**


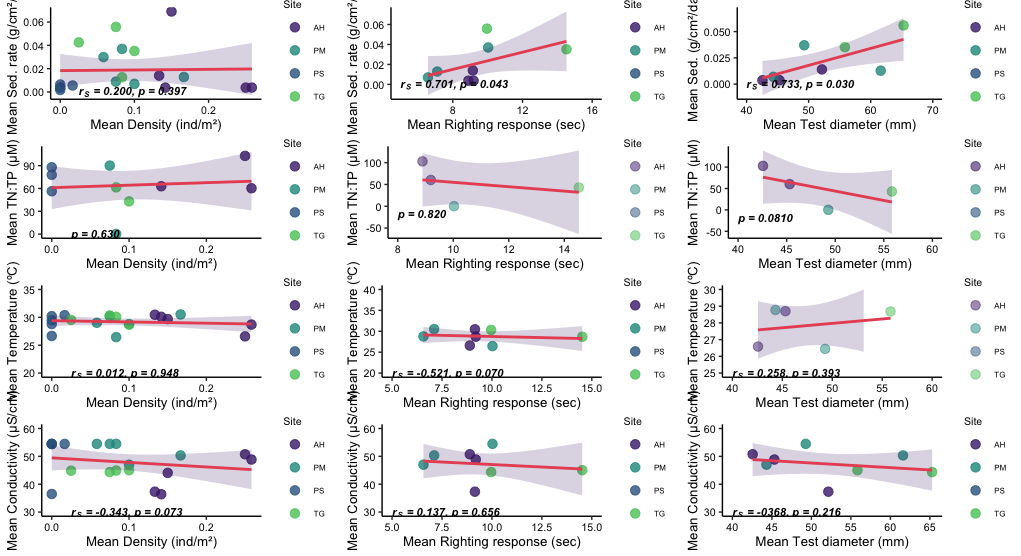


**Figure S1. Environmental correlates of *D. antillarum* physiological metrics (Righting time + Test diameter) and density.** Scatter plots of Euclidean distances in DNA methylation and physiological profiles across seasonal timepoints. Points are colored by site. Solid lines show linear fit; shaded regions denote 95% CI. Spearman’s correlation coefficients (rₛ) and *p-values* are shown.
