# Supplementary material for: Physiological and Epigenetic Responses of the Long‐Spined Sea Urchin Diadema antillarum Across a Spatiotemporal Gradient
Source: Ecol Evol. 2026 Jan 12;16(1):e72915. doi: 10.1002/ece3.72915 (PMC12793898; doi:10.1002/ece3.72915)
Supplement: Supplementary file 2 — Appendix S2: Supporting Information. [file ECE3-16-e72915-s002.docx]

**Supplemental Text**

Text S1……………………………………………………………………………….. 2

**Supplemental Tables**

Table S1……………………………………………………………………………….4

Table S2……………………………………………………………………………….5

Table S3……………………………………………………………………………….6

Table S4……………………………………………………………………………….7

Table S5……………………………………………………………………………….9

**Supplemental Figures**

Figure S1………………………………………………………………………………10

Figure S2………………………………………………………………………………11

Figure S3………………………………………………………………………………12

Figure S4………………………………………………………………………………12

Figure S5………………………………………………………………………………13

Figure S6………………………………………………………………………………14

Figure S7………………………………………………………………………………15

**Supplemental Text**

**Text S1. Protocol for methylation-sensitive amplified polymorphism (MSAP) analysis (see Table S1).**

Genomic DNA underwent concurrent digestion using EcoRI (0.5 U per reaction) in combination with one of two methylation-sensitive isoschizomeric enzymes—either *Hpa*II or *Msp*I (0.2 U per reaction for each)—which differ fundamentally in their sensitivity to DNA methylation (cytosine methylation at CpG sites). *Eco*RI and *Hpa*II/*Msp*I adapters were ligated to resulting fragments. Each reaction contained 200 ng DNA, 0.5 μl *Eco*RI (Thermo Scientific™ FastDigest), 0.2 μl *Hpa*II or *Msp*I (Thermo Scientific™ FastDigest), 1.1 μl Digestion Buffer (10X), 0.55 μl BSA (1 mg/ml), 0.01 U T4 Ligase (Invitrogen™), 1.1 μl Ligase Buffer (10X), 1 μl *Eco*RI adapters (forward and reverse, 5 μM), and 1 μl *Hpa*II/*Msp*I adapters (50 μM). Reactions were incubated at 37 °C for 3 hours. Digestion-ligation PCR products were visualized with gel electrophoresis to confirm the reaction success.

Resulting digested DNA fragments were diluted with 90 μl molecular grade water and subjected to two pre-selective PCR reactions (A and B). Each reaction included 4 μl diluted DNA, 0.2 μl DreamTaq Polymerase, 2 μl Taq Polymerase Buffer (10x), 1.5 μl dNTP Mix (10 μM), 1 μl *Eco*RI+X primer (20 μM), 1 μl HpaII/MspI+X primer (20 μM), 1 μl MgCl2 (25 mM), and 10.5 μl molecular grade water. Pre-selective start with 2 min at 72 °C. After initial denaturation at 94 °C for 2 min, reactions underwent 20 cycles of denaturation (94 °C for 20 sec), annealing (56 °C for 30 sec), and extension (72 °C for 2 min), with a final extension at 60 °C for 30 min. Gel electrophoresis confirmed success.

Pre-selective PCR products were further diluted with 90 μl water and selectively amplified in three additional PCR reactions. Each reaction included 0.5 μl diluted product, 0.2 μl DreamTaq Polymerase, 2 μl Taq Polymerase Buffer (10x), 1.5 μl dNTP Mix (10 μM), 1 μl each fluorescently labeled *Hpa*II/*Msp*I+X primer (8.3 μM) in the selective pair, 1 μl MgCl2 (25 mM), and 14 μl water. After initial denaturation and extension, reactions underwent cycling conditions as described, with selective PCR products confirmed by gel electrophoresis.

Multiplexing of DNA fragments from Primers C1/C2 and C3/C4 was conducted (54.2% C1, 37.5% C2, 8.3% water), followed by combining 2 μl diluted product with 9.5 μl Hi-Di Formamide (ThermoFisher) and 1 μl MapMarker 1500-ROX (BioVentures). Fragment analysis occurred on an ABI Prism 310 Genetic Analyzer at the Florida International University DNA Core Facility.

Below are the PCR profile used:

| **Pre-Selective** | | | |
| --- | --- | --- | --- |
|  | 72°C | 2min |  |
| Denaturing | 94°C | 20 sec | X 20 Cycles |
| Annealing | 56°C | 30 sec |  |
| Extension | 72°C | 2 min |  |
|  | 60°C | 30 min |  |
| **Selective:** | | | |
|  | 72°C | 2min |  |
| Denaturing | 94°C | 20 sec | X 20 Cycles |
| Annealing | 66°C | 30 sec |  |
| Extension | 72°C | 2 min |  |
| Denaturing | 94°C | 20 sec | X 20 Cycles |
| Annealing | 56°C | 30 sec |  |
| Extension | 72°C | 2 min |  |
|  | 60°C | 30 min |  |

**Supplemental Tables**

**Table S1. MSAP adapter and primer sequences.** Nucleotide sequences (5′–3′) of adapters and primers used at each stage of the MSAP protocol, including digestion-ligation, pre-selective PCR (A or B), and selective PCR (C1, C2, C3, or C4). Selective PCR primers were fluorescently labeled with either FAM or HEX dyes, as indicated by an asterisk.

| **Step** | **Oligo type** | **Sequence** |
| --- | --- | --- |
| Digestion-ligation | EcoRI forward adapter  EcoRI reverse adapter  HpaII/MspI forward adapter  HpaII/MspI reverse adapter | CTCGT AGA CTG CGT ACC  AATTG GTA CGC AGT CTAC  CGTTC TAG ACT CATC  GACGA TGA GTC TAGAA |
| Pre-selective PCR A | EcoRI + A  HpaII/MspI + T | GACTG CGT ACC AAT TCA  GATGA GTC TAG AAC GGT |
| Pre-selective PCR B | EcoRI + C  HpaII/MspI + A | GACTG CGT ACC AAT TCC  GATGA GTC TAG AAC GGA |
| Selective PCR C1 | HpaII/MspI + TGC (*HEX)  HpaII/MspI + TAA (*HEX) | GATGA GTC TAG AAC GGTGC GATGA GTC TAG AAC GGTAA |
| Selective PCR C2 | HpaII/MspI + TTG (*FAM) HpaII/MspI + TCT (*FAM) | GATGA GTC TAG AAC GGTTG  GATGA GTC TAG AAC GGTCT |
| Selective PCR C3 | HpaII/MspI + AGT (*HEX) HpaII/MspI + ACG (*HEX) | GATGA GTC TAG AAC GGAGT  GATGA GTC TAG AAC GGACG |
| Selective PCR C4 | HpaII/MspI + ATG (*FAM) HpaII/MspI + AGA (*FAM) | GACTG CGT ACC AAT TCATG  GACTG CGT ACC AAT TCAGA |

**Table S2. Pairwise comparisons of *Diadema antillarum* A) density and B) Survival rate of transplanted urchins, across sites using a General Linear Model with Poisson distribution (AIC = 335.14).** Adjusted *p-values* from *Tukey’s HSD* tests are shown on the above diagonal. Significant values (α = 0.05) are bolded; NA indicates non-significant comparisons not tested. The asterisks (*) indicate the level of significance.

| **A1. GLM with Poison distribution** | | | | | | | | | | |
| --- | --- | --- | --- | --- | --- | --- | --- | --- | --- | --- |
| **Sites** | **Estimate** | | **Std Error** | | **z value** | | **Pr (>\|z\|)** | |  | |
| (Intercept) | *3.0845* | | *0.0808* | | *38.154* | | ***< 2x10^-16^*** | | ********* | |
| TG | *-0.7033* | | *0.1222* | | *-5.754* | | ***8.71x10^-9^*** | | ***** | |
| PM | *-0.4454* | | *0.1243* | | *-3.582* | | ***0.0003*** | | ***** | |
| PS | *-1.1386* | | *0.1397* | | *-8.149* | | ***3.67x10^-16^*** | | ***** | |
|  | | | | | | | | | | |
| **A2. Tukey comparison** | | | | | | | | | |  |
|  | | **AH** | | **TG** | | **PM** | | **PS** | |  |
| **AH** | |  | | *0.0705* | | *0.4276* | | ***0.0032*** | |  |
| **TG** | | *NA* | |  | | *0.8407* | | *0.5885* | |  |
| **PM** | | *NA* | | *NA* | |  | | *0.1947* | |  |
| **PS** | | *NA* | | *NA* | | NA | |  | |  |
|  | | | | | | | | | | |
| **B. GLM with binomial family** | | | | | | | | | | |
| **Sites** | **Estimate** | | **Std Error** | | **z value** | | **Pr (>\|z\|)** | |  | |
| (Intercept) | 20.9713 | | 3180.2431 | | 0.006 | | 0.995 | |  | |
| TG | *-0.4292* | | *0.7802* | | *-0.550* | | 0.582 | |  | |
| PSC | 1.3275 | | 1.2508 | | 1.061 | | 0.289 | |  | |
| PS | *-0.1325* | | *0.7782* | | *-0.170* | | 0.865 | |  | |
| Status:DA | *-19.8804* | | *3280.2430* | | *-0.006* | | *0.995* | |  | |
| Status:AA | *-*20.2606 | | *3280.2431* | | *-0.006* | | *0.995* | |  | |

**Table S3.** **Pairwise PERMANOVA comparisons of physiological performance metrics (righting response and test diameter) in *D. antillarum* across environmental factors.** (A) Monitored urchins; (B) Transplanted urchins. Upper diagonal shows PERMANOVA *p-values* (*pairwise.adonis* function); lower diagonal shows dispersion comparisons (*TukeyHSD* function). NA = comparison not performed due to non-significance. Significant values (α = 0.05) are bolded.

| **A. Monitored** | | | | |
| --- | --- | --- | --- | --- |
| **A1. Site** | | | | |
|  | **AH** | **TG** | **PM** |  |
| **AH** |  | ***0.006*** | *0.702* |  |
| **TG** | *NA* |  | ***0.003*** |  |
| **PM** | *NA* | *NA* |  |  |
|  | | | | |
| **A2. Season** | | | | |
|  | **Dry** | **Wet** |  |  |
| **Dry** |  | ***0.031*** |  |  |
| **Wet** | *NA* |  |  |  |
|  | | | | |
| **B. Transplanted** | | | | |
| **B1. Site** | | | | |
|  | **TG** | **PS** | **TGC** | **PSC** |
| ***TG*** |  | ***0.024*** | ***0.012*** | ***0.030*** |
| ***PS*** | *NA* |  | ***0.006*** | ***0.036*** |
| ***TGC*** | *NA* | *NA* |  | ***0.006*** |
| ***PSC*** | *NA* | *NA* | *NA* |  |
|  | | | | |
| **B2. Status** | | | | |
|  | **BT** | **DA** | **AA** |  |
| **BT** |  | *1.000* | *0.924* |  |
| **DA** | *NA* |  | *0.495* |  |
| **AA** | *NA* | *NA* |  |  |

Note: TGC and PSC correspond to the controls in each site.

**Table S4. Pairwise comparisons of righting response and test diameter for (1) Monitoring *D. antillarum* across environmental factors (site and season) and (2) transplanted *D. antillarum* by site and transplant status.** Estimated marginal means (*emmeans* function) with significant *p-values* (α = 0.05) in bold. NA indicates comparisons omitted due to interactions or lack of effect (see Supplemental repository data **Tables S4** and Supplemental material **Table S2**).

| **1. Monitored D. antillarum (11-month period)** | | | | |
| --- | --- | --- | --- | --- |
| **A. Righting response (seconds)** | | | | |
| **A.1 Site** | | | | |
|  | **TG** | **PM** | **AH** |  |
| **TG** |  | ***0.0002*** | ***0.0068*** |  |
| **PM** | *NA* |  | *0.4744* |  |
| **AH** | *NA* | *NA* |  |  |
|  | | | | |
| **A.2 Season** | | | | |
|  | **Dry** | **Wet** |  |  |
| **Dry** |  | ***0.009*** |  |  |
| **Wet** | *NA* |  |  |  |
|  | | | | |
| **B. Test diameter (mm)** | | | | |
| **B.1 Site** | | | | |
|  | **TG** | **PM** | **AH** |  |
| **TG** |  | ***<0.0001*** | ***<0.0001*** |  |
| **PM** | *NA* |  | *0.7745* |  |
| **AH** | ***NA*** | ***NA*** |  |  |
|  | | | | |
| **B.2 Season** | | | | |
|  | **Dry** | **Wet** |  |  |
| **Dry** |  | *0.9383* |  |  |
| **Wet** | *NA* |  |  |  |
|  | | | | |
| **2. Transplanted *D. antillarum* (3-months experiment period)** | | | | |
| **A. Righting response (seconds)** | | | | |
| **A.1 Site** | | | | |
|  | **TG** | **PS** | **TGC** | **PSC** |
| **TG** |  | ***0.0007*** | *0.9888* | *0.9993* |
| **PS** | *NA* |  | *0.0956* | ***0.0419*** |
| **TGC** | *NA* | *NA* |  | 0.9984 |
| **PSC** | *NA* | *NA* | ***NA*** |  |
|  | | | | |
| **A.2 Status** | | | | |
|  | **BT** | **DA** | **AA** |  |
| **BT** |  | *0.4062* | *0.8546* |  |
| **DA** | *NA* |  | *0.7243* |  |
| **AA** | *NA* | *NA* |  |  |
|  | | | | |
| **B. Test diameter (mm)** | | | | |
| **B.1 Site** | | | | |
|  | **TG** | **PS** | **TGC** | **PSC** |
| **TG** |  | *1.000* | ***0.0294*** | *0.0784* |
| **PS** | *NA* |  | ***0.0278*** | *0.0747* |
| **TGC** | *NA* | *NA* |  | *0.9722* |
| **PSC** | *NA* | *NA* | *NA* |  |
|  | | | | |
| **B.2 Status** | | | | |
|  | **BT** | **DA** | **AA** |  |
| **BT** |  | *0.9446* | *0.7701* |  |
| **DA** | *NA* |  | *0.9304* |  |
| **AA** | *NA* | NA | NA |  |

**Table S5. Pairwise PERMANOVA comparisons of DNA methylation patterns in *D. antillarum*. (A) Monitored urchins; (B) Transplanted urchins.** Upper diagonal: PERMANOVA *p-values* (*pairwise.adonis* function). Lower diagonal: *TukeyHSD* comparisons of dispersion. Significant p-values (α = 0.05) are bolded; NA = comparison not performed due to lack of significance.

| **A. Monitored urchins** | | | | |
| --- | --- | --- | --- | --- |
| **A.1 Site** | | | | |
|  | **TG** | **PM** | **AH** |  |
| **TG** |  | *NA* | *NA* |  |
| **PM** | *0.594* |  | *NA* |  |
| **AH** | ***0.033*** | ***0.003*** |  |  |
|  | | | | |
| **A.2 Season** | | | | |
|  | **Dry** | **Wet** |  |  |
| **Dry** |  | *0.001* |  |  |
| **Wet** | *NA* |  |  |  |
|  | | | | |
| **B. Transplanted urchins** | | | | |
| **B.1 Site** | | | | |
|  | **TG** | **PS** | **TGC** | **PSC** |
| **TG** |  | ***0.018*** | ***0.006*** | ***0.030*** |
| **PS** | *NA* |  | ***0.006*** | ***0.042*** |
| **TGC** | *NA* | *NA* |  | ***0.006*** |
| **PSC** | *NA* | *NA* | *NA* |  |
|  | | | | |
| **B.2 Status** | | | | |
|  | **BT** | **DA** | **AA** |  |
| **BT** |  | *1.000* | *0.849* |  |
| **DA** | *NA* |  | *0.492* |  |
| **AA** | *NA* | *NA* |  |  |

**Supplemental Figures and Captions**

**
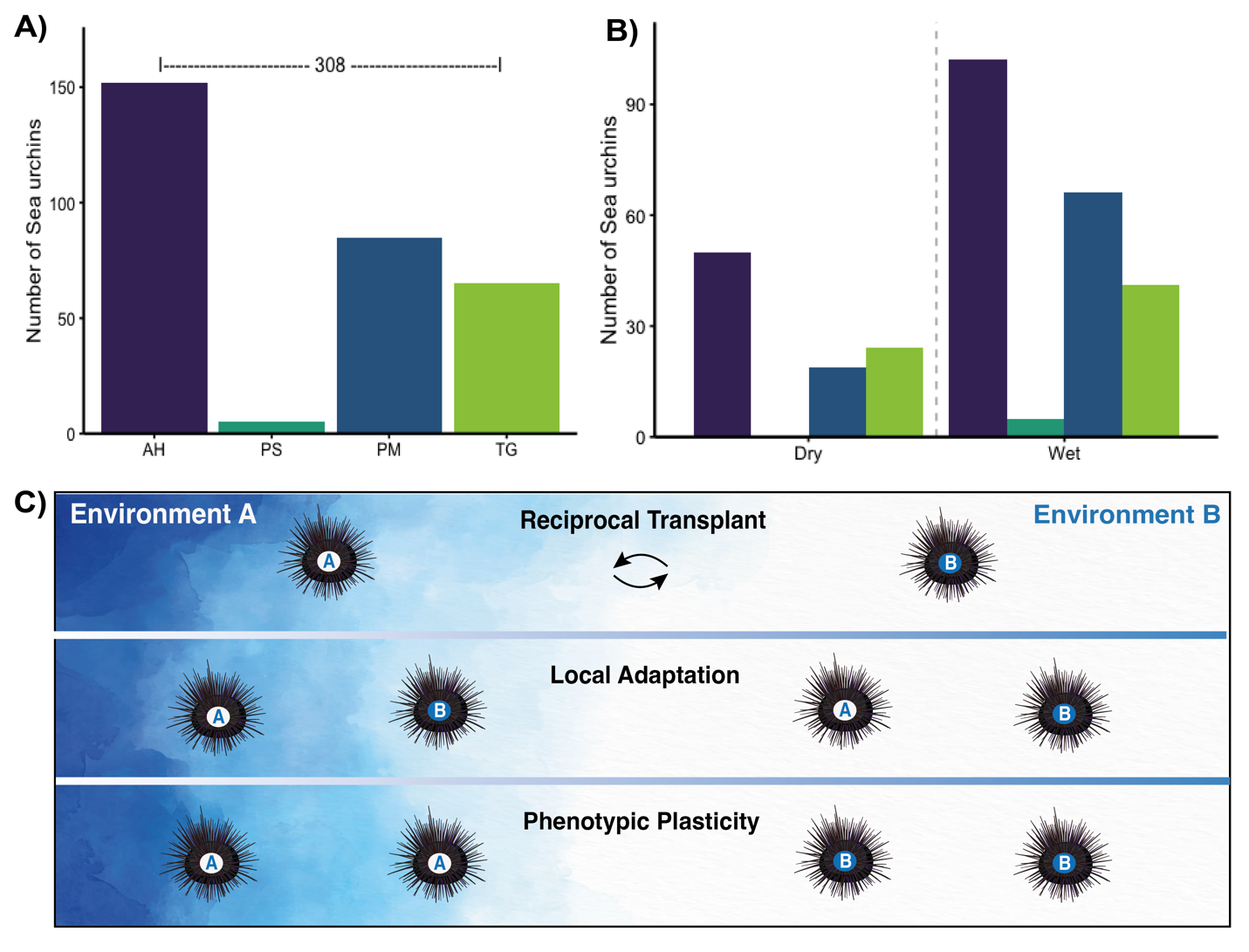
**

**Figure S1. Sampling effort and experimental design. (A)** Number of individuals surveyed per site during monitoring period. **(B)** Individuals surveyed by season in the monitoring period. **(C)** Experimental design diagram illustrating reciprocal transplants of *D. antillarum* between contrasting environments to test for local adaptation and plasticity.

**
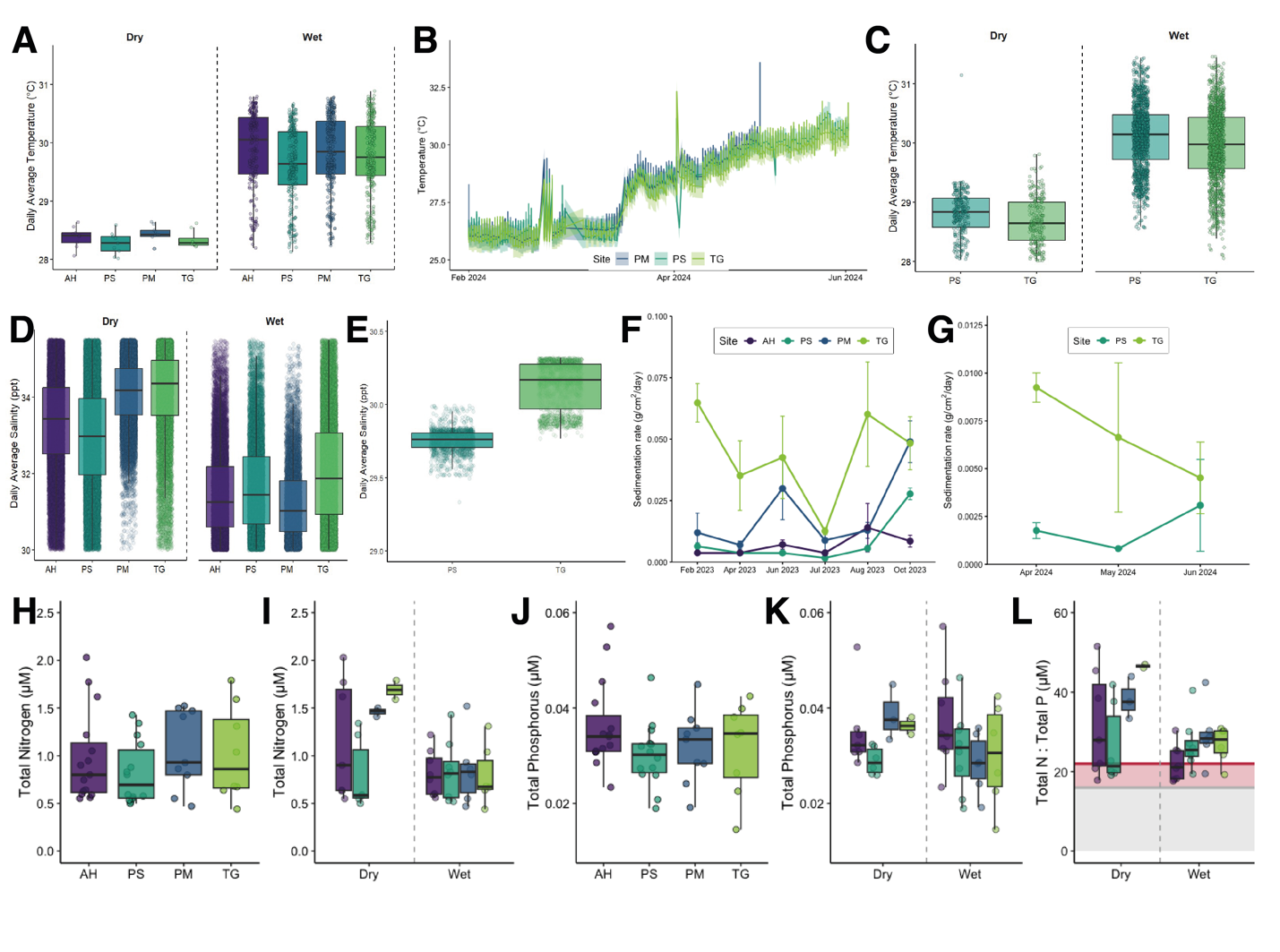
**

**Figure S2. Additional environmental conditions across sites and seasons. (A)** Daily standard deviations of temperature (°C) during 2023 monitoring. (**B)** Temporal evolution of water temperature (°C) throughout the transplant period across all study sites. Solid lines represent daily mean values, while the surrounding shaded envelopes delineate the diurnal thermal range (daily minima and maxima). The synchronized pattern across sites reveals consistent seasonal thermal progressions with site-specific variability in thermal amplitude. (**C)** Daily standard deviations of temperature (°C) during transplant period. (**D)** Daily standard deviations of salinity by site and season during 2023 monitoring. (**E)** Daily standard deviations of salinity by site and season during transplant period. (**F-G)** Sedimentation rate (g/cm²/day) by site during 2023 monitoring (1) and during the transplant perido (2). **(H-L)** Nutrient concentrations and N:P ratio by sites across seasons. Boxplots display summary statistics; individual data points are overlaid.

**
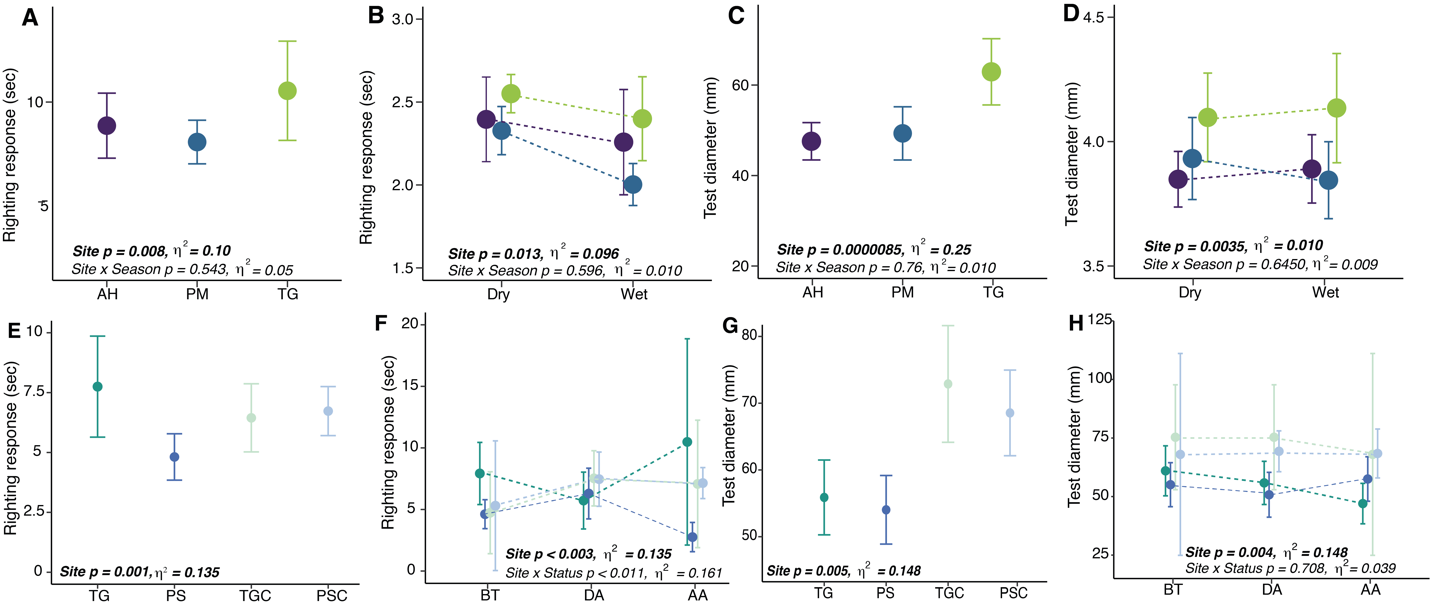
Figure S3. Reaction norms of *D. antillarum* physiological traits. (A–B, E-F)** Righting response (seconds) and test diameter (mm) by site. **(C–D, H-I)**. Same variables across seasonal timepoints.
Colored lines represent site-level trends. Mean ± 95% CI shown. Model *p-values* and η² effect sizes (*anova* and *eta_squared* functions, respectively) from linear models are indicated (supplemental repository data **Table S7**).

**
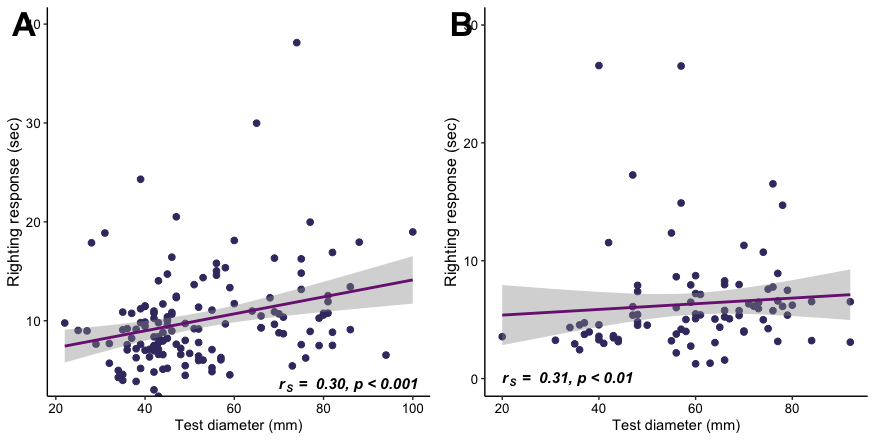
**

**Figure S4. Correlation between Righting time and Test diameter. A)** Monitored urchins. **B)** Transplanted urchins.


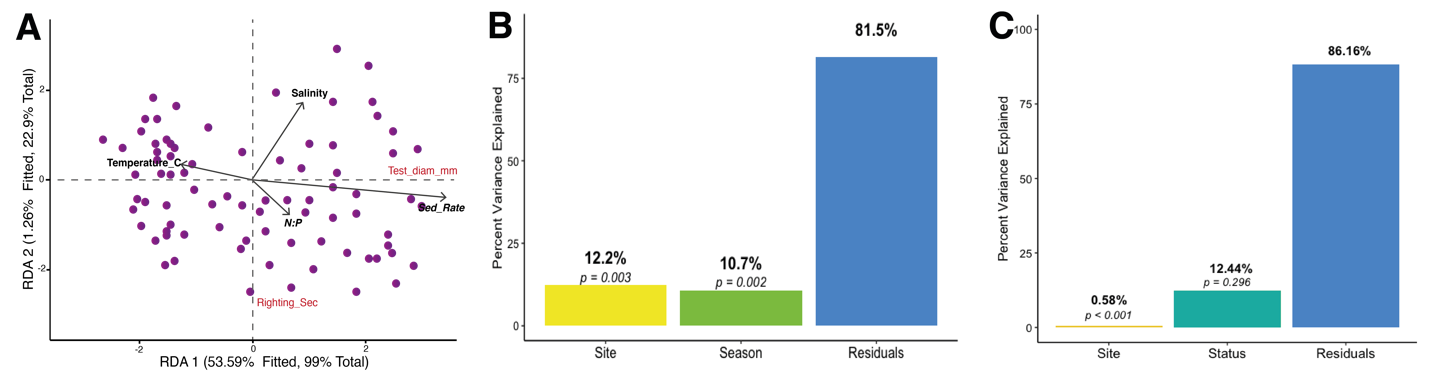


**Figure S5.** **Environmental drivers of physiological performance (Righting time + Test diameter) variation in urchin populations**. (A) Multivariate redundancy analysis (RDA) illustrating the relationship between environmental conditions and physiological metrics. Individual data points represent the comprehensive physiological profile of each specimen, with vectors indicating the direction and magnitude of influence for significant environmental parameters (see Table S8). (B) Variance partitioning analysis quantifying the relative contributions of spatial (yellow) and temporal/seasonal (green) factors to the observed variation in multivariate urchin physiology, with residual unexplained variance indicated in blue. Statistical significance (*p-values*) for each predictor variable is provided based on partial redundancy analysis (RDA), controlling for covariate effects. (C) Multivariate physiological response partitioning in transplanted populations. Variance decomposition analysis of multivariate physiological responses during the transplantation experiment. Proportional contributions to physiological variation are quantified for spatial factors (yellow), temporal/seasonal influences (tail), and residual unexplained variance (blue). Statistical significance (*p-values*) for individual predictors is determined through partial redundancy analysis (RDA), controlling for potential confounding variables.

**
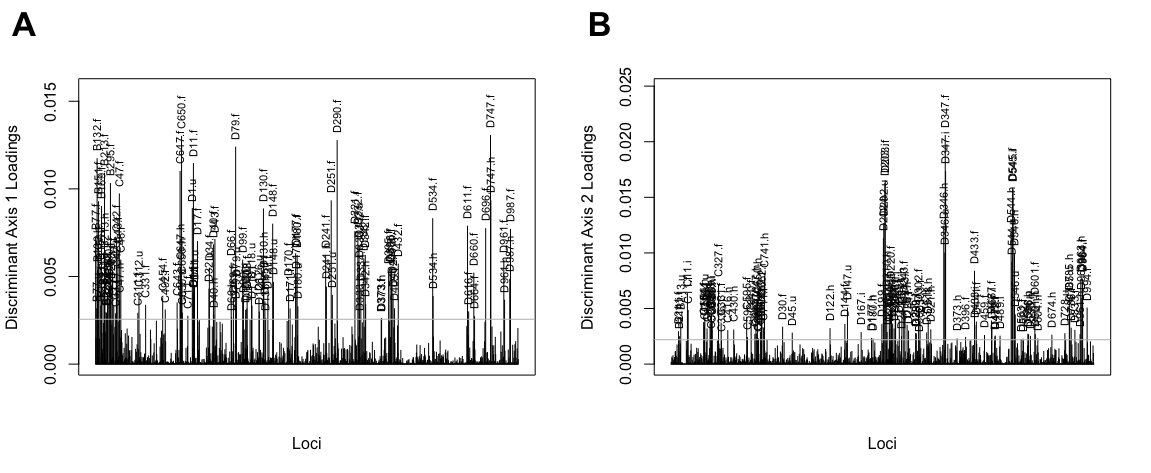
**

**
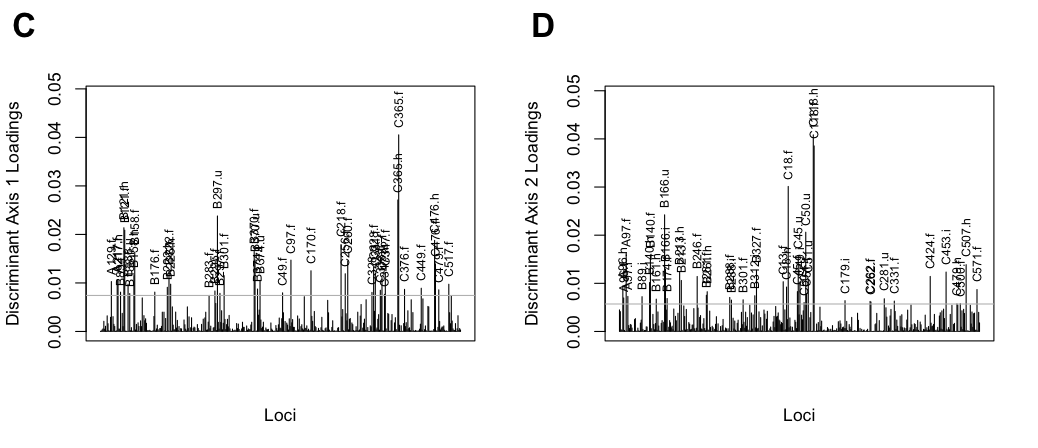
**

**Figure S6. Loading plots from the Discriminant Analysis of Principal Components (DAPC) of DNA methylation profiles of** **(A–B) Monitored urchins; (C–D) Transplanted urchins.** The loadings of loci and DNA methylation states across the first (A and C) and second (B and D) discriminant axes within the DAPC analysis of DNA methylation profiles of each monitoring and transplant are shown. Loci with loading scores in the 90^th^ percentile are labeled and identified as the most influential loci.

**
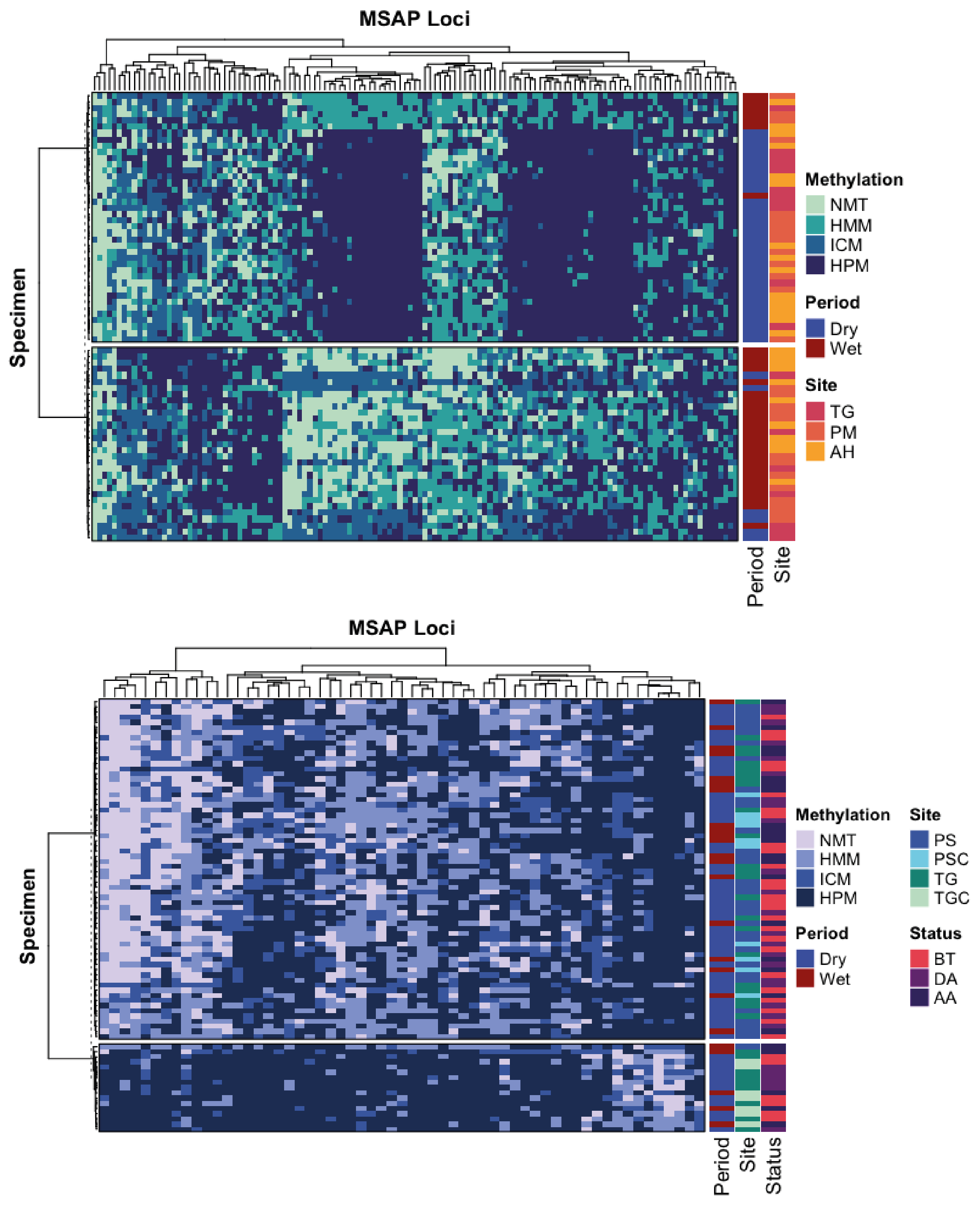
**

**Figure S7. Heatmap of methylation states in *D. antillarum* (A) Monitored and (B) Transplanted individuals.** Categorical methylation state (NMT: non-methylated, HMM: hemi-methylated, ICM: methylated at an internal cytosine, or HPM: hypermethylation) of the most influential **(Figure S6)**. Hierarchical clustering and k-means grouping are shown, with grouping factors (site, season) annotated. Heatmap generated using ComplexHeatmap (Gu et al., 2016).

**Reference:**

Gu, Z., Eils, R., & Schlesner, M. (2016). Complex heatmaps reveal patterns and correlations in multidimensional genomic data. *Bioinformatics (Oxford, England)*, *32*(18), 2847–2849.
